# Targeting extracellular matrix through phytochemicals: a promising approach of multi-step actions on the treatment and prevention of cancer

**DOI:** 10.3389/fphar.2023.1186712

**Published:** 2023-07-25

**Authors:** Dan Liang, Lu Liu, Yunjie Zhao, Zhenyi Luo, Yadi He, Yanping Li, Shiyun Tang, Jianyuan Tang, Nianzhi Chen

**Affiliations:** ^1^ Hospital of Chengdu University of Traditional Chinese Medicine, Chengdu, China; ^2^ College of Pharmacy, Chengdu University of Traditional Chinese Medicine, Chengdu, China; ^3^ Key Laboratory of Marine Fishery Resources Exploitment and Utilization of Zhejiang Province, College of Pharmaceutical Science and Collaborative Innovation Center of Yangtze River Delta Region Green Pharmaceuticals, Zhejiang University of Technology, Hangzhou, China; ^4^ Graduate School, Guangxi University of Chinese Medicine, Nanning, China; ^5^ College of Acupuncture and Tuina, Chengdu University of Traditional Chinese Medicine, Chengdu, China; ^6^ School of Basic Medical Sciences, Chengdu University of Traditional Chinese Medicine, Chengdu, China; ^7^ State Key Laboratory of Ultrasound in Medicine and Engineering, College of Biomedical Engineering, Chongqing Medical University, Chongqing, China

**Keywords:** TME, tumor microenvironment, ECM, extracellular matrix, CAFs, cancer-associated fibroblasts, TAMs, tumor-associated macrophages, HA, hyaluronic acid, MMPs, matrix metalloproteinases, HSPGs, heparan sulfate proteoglycans, TGFβ, transforming growth factor beta

## Abstract

Extracellular matrix (ECM) plays a pivotal and dynamic role in the construction of tumor microenvironment (TME), becoming the focus in cancer research and treatment. Multiple cell signaling in ECM remodeling contribute to uncontrolled proliferation, metastasis, immune evasion and drug resistance of cancer. Targeting trilogy of ECM remodeling could be a new strategy during the early-, middle-, advanced-stages of cancer and overcoming drug resistance. Currently nearly 60% of the alternative anticancer drugs are derived from natural products or active ingredients or structural analogs isolated from plants. According to the characteristics of ECM, this manuscript proposes three phases of whole-process management of cancer, including prevention of cancer development in the early stage of cancer (Phase I); prevent the metastasis of tumor in the middle stage of cancer (Phase II); provide a novel method in the use of immunotherapy for advanced cancer (Phase III), and present novel insights on the contribution of natural products use as innovative strategies to exert anticancer effects by targeting components in ECM. Herein, we focus on trilogy of ECM remodeling and the interaction among ECM, cancer-associated fibroblasts (CAFs) and tumor-associated macrophages (TAMs), and sort out the intervention effects of natural products on the ECM and related targets in the tumor progression, provide a reference for the development of new drugs against tumor metastasis and recurrence.

## Graphical Abstract

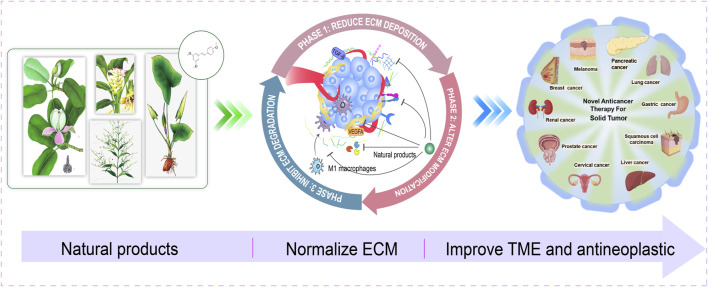

## Highlights

1 Based on extensive researches on extracellular matrix (ECM), this review provides a reference for the subsequent research on the mechanism of tumor occurrence and development and the development of new drug targets in the future.
2 Based on the coincidence between the whole-process of tumor progression and the ECM remodeling trilogy, we propose three phases of whole-process management of cancer and the necessity of targeting ECM remodeling trilogy in the treatment of tumors.
3 This paper is the first time comprehensively to summarize the current situation of natural products targeting ECM research, providing more new perspectives and research fields for following researchers.

## 1 Introduction

Statistics showed that there were 19.3 million new cancer diagnoses and 10 million new deaths worldwide in 2020. The cancer burden is predicted to reach 28.4 million by 2040, a 47% increase from 2020 (Sung et al., 2021). Malignant tumors spring up with high morbidity and mortality but lack of effective therapies (Huang et al., 2020; Tiwari et al., 2022). Current systemic antitumor therapy still has a high probability of tumor recurrence and distant metastasis (D'Alterio et al., 2020; Basu et al., 2022). Immune escape, early metastasis, and drug resistance under tumor microenvironment (TME) protection are the main reasons for the frustration of cancer therapy (Senthebane et al., 2017; Chen and Dey, 2022). There are dynamic interactions between cellular and non-cellular components in the TME. Their constant variations contribute to the generation of tumor cell heterogeneity, clonal evolution, enhanced multidrug resistance of tumor cells, and ineffectiveness of immunotherapy (Dzobo et al., 2023). Reprogramming of TME components is one of the possible exits. Coincidentally, extracellular matrix (ECM) is the major non-cellular component of TME, gradually becoming a hotspot in tumor therapy (Walker et al., 2018; Karamanos et al., 2021).

ECM is an insoluble structural component of stroma in mesenchyma and epithelial blood vessels, including collagen, elastin, proteoglycan and glycoprotein (Chaudhuri et al., 2020). It interacts with tumor and stromal cells to trigger uncontrolled cell proliferation, migration, invasion and immune evasion of during the lengthy process of precancerous lesions progressing to malignancy (Pickup et al., 2014). This whole process is accompanied by variations in the ECM’s own structure.

ECM remodeling includes the deposition of cells and components in the ECM, the abnormal modification caused by increasing ECM-modifying enzymes, and the degradation of ECM conducted by proteases (Winkler et al., 2020). The dysregulation of the above mechanisms, especially the amount and cross-linking state of ECM components, induce ECM stiffness variation (Meng et al., 2016; Hua et al., 2020; Hua et al., 2021; Tzanakakis et al., 2021; Yang et al., 2021). ECM stiffness and ECM degradation are the main contributors to cancer metastasis which also contribute to the deterioration of tumor (Najafi et al., 2019). The trilogy of ECM remodeling are exist simultaneously throughout the tumor progression cycle, with different emphases occurring in the early-, middle-, and advanced-stages.

**FIGURE 1:**
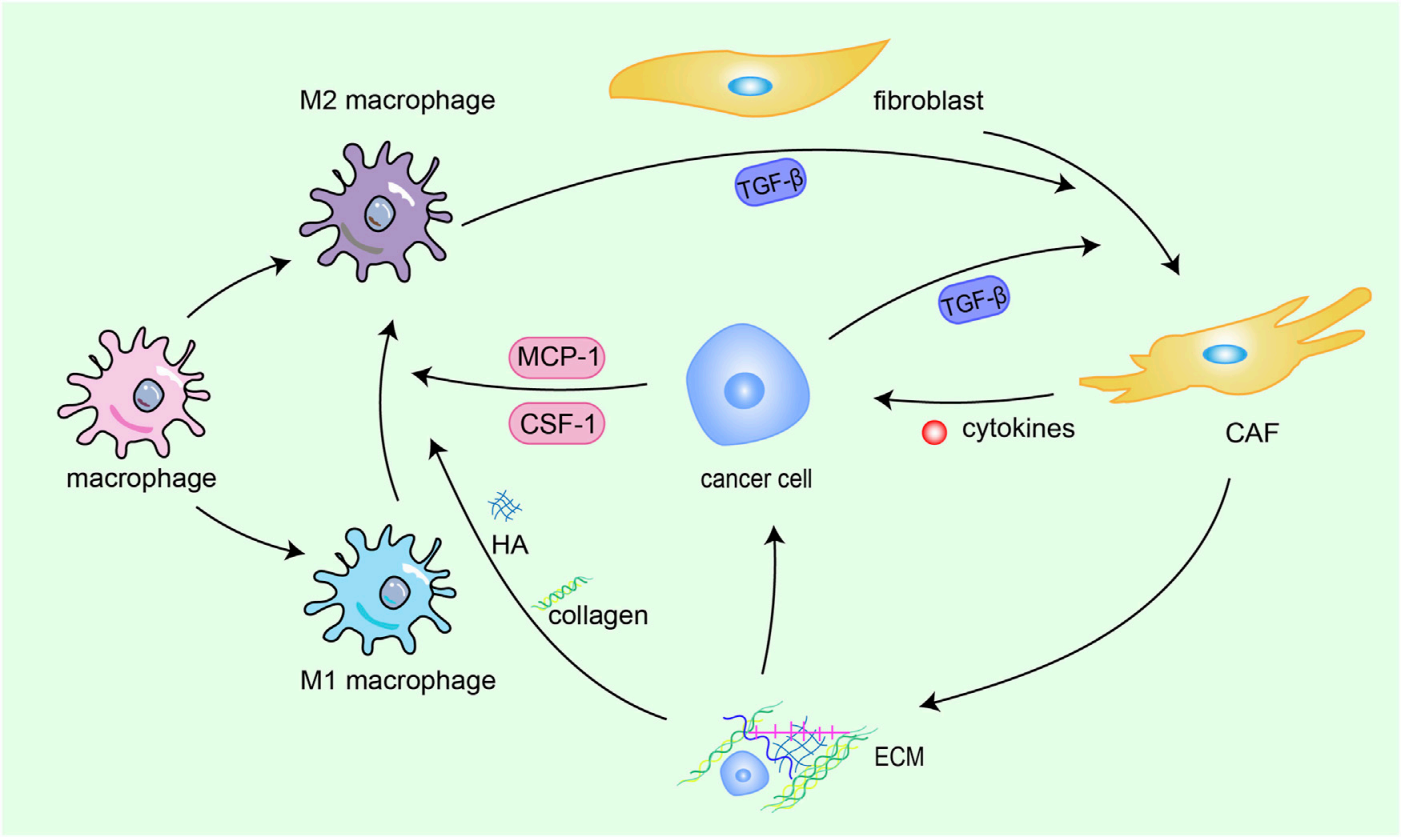
Crosstalk among CAFs, TAMs, ECM and cancer cells. Fibroblasts are attracted to migrate toward the TME by TGF-β derived from cancer cells and M2 macrophages, then switch into CAFs with greater abilities to proliferate and promote ECM accumulation. CAFs promote the synthesis of collagen and fibronectin and chemokines related to tumor promotion, which heighten cancer cell invasion and ultimately contribute to the occurrence of organ-specific metastases. This interaction between cancer cells and CAFs constitute s a forward circulation that fuels rapid tumor development. TAMs are constantly affected by physical, chemical, and biological signals from cancer cells and the ECM. ECM components drive M2 polarization. TAMs can weave an immunosuppressive network by secreting immunosuppressive factors, providing more fuel to this vicious cycle, thereby promoting the activation of CAFs and leading to malignant transformation.

In addition, the signaling loop formed by TAMs, CAFs and tumor cells contribute to ECM remodeling. Fibroblasts, the fundamental cell types in the TME and the producer of ECM, play a pivotal role in the construction and remodeling of the ECM (Kalluri and Zeisberg, 2006). Fibroblasts are attracted to migrate toward the TME by transforming growth factor beta (TGF-β) derived from cancer cells, then switch into CAFs with greater abilities to proliferate and promote ECM accumulation (Voloshenyuk et al., 2011; Terra et al., 2018). CAFs promote the synthesis of collagen and fibronectin and chemokines related to tumor promotion (Ignotz and Massagué, 1986; Kuzet and Gaggioli, 2016), which heighten cancer cell invasion and ultimately contribute to the occurrence of organ-specific metastases. This interaction between cancer cells and CAFs constitute s a forward circulation that fuels rapid tumor development. TAMs are the most common immune cells in the TME, and TAMs are constantly affected by physical, chemical, and biological signals from cancer cells and the ECM (Tajaldini et al., 2022). ECM components can drive M2 polarization (Tariq et al., 2017), TAMs can weave an immunosuppressive network by secreting immunosuppressive factors are released into the TME, providing more fuel to this vicious cycle, thereby promoting the activation of CAFs and leading to malignant transformation (Figure 1).

Since the ECM remodeling trilogy exhibits different emphases at different stages, the focus of pharmacotherapy in tumor sequential treatment should be coincident with different emphases at different stages. Thus, we put forward the concept of three phases of the whole-process management of cancer around ECM, controlling three phases of cancer progression is key to reducing cancer-related deaths. Phase I: Reducing ECM deposition in an early-stage contribute to improve ECM stiffness and delay the cancerization, such as fibrillation, which effectively reverse the carcinogenesis of precancerous lesions. Targeting MMPs can theoretically inhibit the likelihood of early metastasis. Phase II: During the middle stages of cancer progression, reversing the tumorigenic ECM modification and inhibiting ECM degradation can decrease the possibility of advanced cancer metastasis and recurrence. Inhibiting ECM deposition facilitates chemotherapy drugs reach the inside of the tumor through dense ECM, so that improving clinical efficacy. Phase III: In the advanced stages, regulating ECM degradation is beneficial to reduce tumor neovascularization, alleviate widespread metastasis and facilitate therapeutic drugs enter the dense microenvironment of solid tumors, such as pancreatic cancer, etc., thereby improving quality of life.

In the past decade, the invention and application of advanced technologies such as large-scale rapid screening, combinatorial chemistry, and genetic engineering have accelerated the process of drug development. The research and development of antineoplastic drugs has entered a new era. Nature is the richest source of structurally novel anticancer drug candidates. Natural products and drugs that are derivatives or mimic natural products and their pharmacophores account for a considerable proportion of anticancer drugs (Mokbel et al., 2019; Atanasov et al., 2021), served as a huge treasure trove of medicines can prepare for ECM preclinical research and new drug development. Clinical practice has shown that active ingredients extracted from natural products such as medicinal plants and Marine organisms, such as flavonoids, terpenoids, alkaloids, polysaccharides, essential oils and peptides, effectively inhibit the growth of tumor cells (Deng et al., 2020; Dong et al., 2022). Many natural products have small side effects and high safety, and could be used as dietary supplements to regulate ECM and curb the occurrence of cancer. There are also problems such as low solubility, low bioavailability and low tumor targeting, leading to the necessity of using large doses of most natural products, which increases the risk of side effects. Nanodrug delivery systems (liposomes, micelles and nanoparticles) provide a powerful backup for the design of advanced drug delivery systems for natural drugs due to their excellent tumor targeting, anti-tumor activity, immune sensitization, stability and safety. Study has shown the inhibitory effect of chitosan nanoparticles enriched with limonene essential oil on melanoma and breast cancer cells (Alipanah et al., 2021).

Currently, there have been reviews of natural products targeting CAF, whereas ECM remodeling is a core transit station in the TME. It’s necessary to review the natural products targeting ECM remodeling and help achieve the whole-process management of cancer. In cancer cell-centric TMEs, regulating angiogenesis, tumor metastasis, and drug resistance by manipulating the composition and structure of the ECM. Natural products can modulate the ECM, cut off cellular crosstalk between CAFs, cancer cells and TAMs, and regulate ECM remodeling to normalize it to inhibit angiogenesis, thereby meliorating the delivery of chemotherapeutic drugs to cancer cells.

## 2 Trilogy of tumorigenic ECM remodeling

**FIGURE 2:**
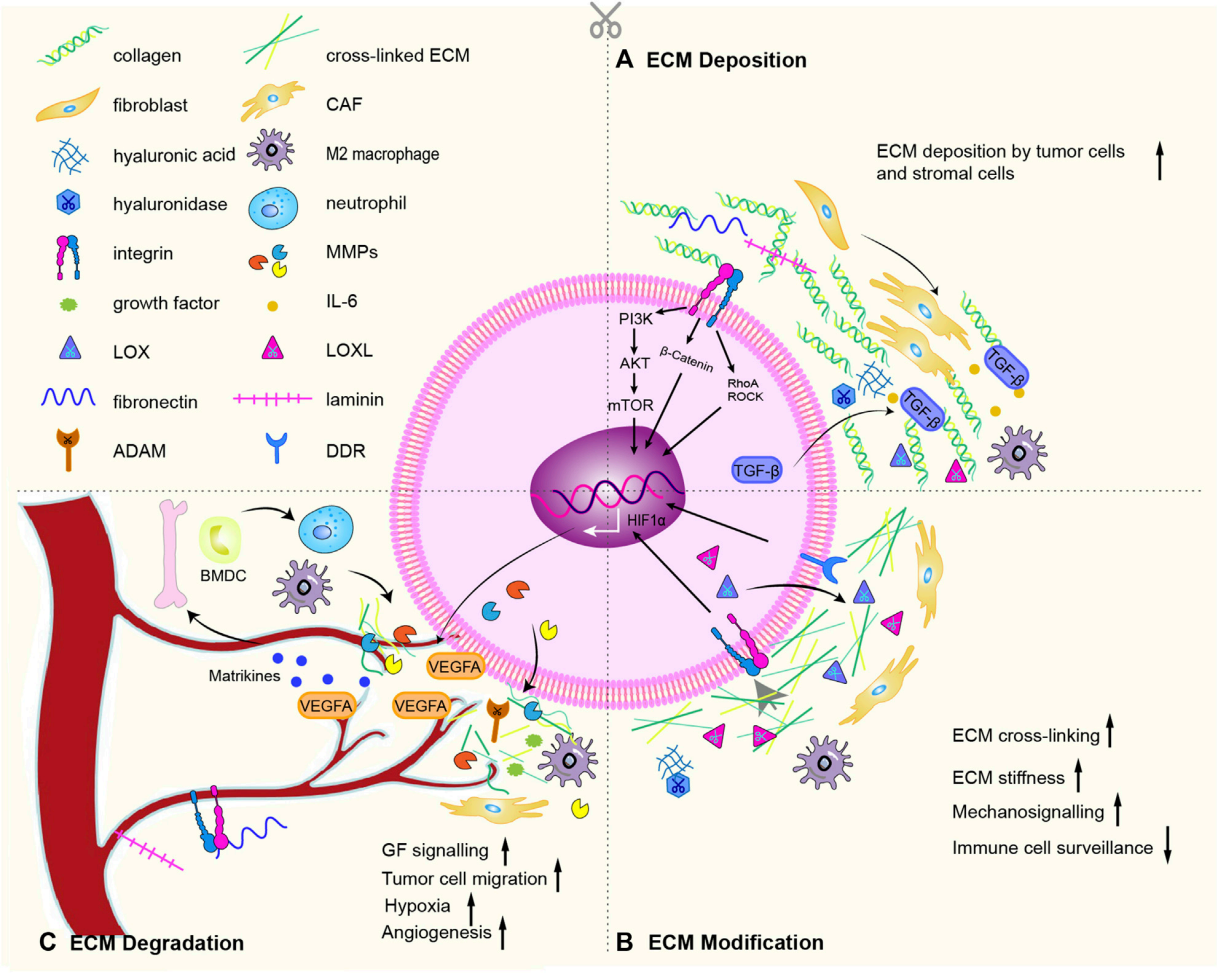
ECM remodeling mechanism. **(A)** Especially through active TGF-β signaling, CAFs deposit abundant collagen and express cytokines, promotes the recruitment and activation of M2 macrophages. The deposition of cells inevitably lead to deposition of ECM subassembly, including collagen, ECM modifying enzymes (LOX, LOX-like protein), fibronectin, hyaluronic acid, tenascin C and laminin. **(B)** Chemical modification alters the biochemical properties and structural characteristics of the ECM. The main proteins involved are LOX, LOXLs, and TG2. Force-mediated ECM remodeling affects ECM tissue by aligning ECM fibers and opening cell migration channels. The main mechanical sensors involved are integrins, ROCK, DDR, Piezo1, YAP/TAZ. Overexpression of LOX and LOXLs increases fibrosis and ECM stiffness, and promotes tumorigenesis and metastasis. During this modification, mechanical forces exerted by aggregated integrins would result in nonproteolytic destruction of the basement membrane, allowing invasion of cancer cells. **(C)** CAFs, cancer cells and recruited BMDC secrete proteases that degrade the ECM: such as MMPs, disintegrins and ADAMs. ECM degradation is an important driver of cancer cell motility. The binding of soluble signaling molecules such as growth factors to the ECM renders them insoluble and inactive, while proteases enables their release. Proteolytic ECM degradation produces bioactive matrikines and releases matrix-bound matrikines. These factors induce pro-tumor ECM signaling that promotes tumor proliferation, migration, invasion, and angiogenesis. Matrikines also induce the activation of BMDC to secrete neutrophils. Neutrophils secrete potent MMP-9, which degrades ECM and releases matrix-bound VEGF, creating a concentration gradient of new angiogenic sprouts. Finally, upon stimulation of dense ECM, tumor cells may acquire endothelial-like functions and mimic the vasculature connected to blood vessels. Some graphic elements refer to published literature (Winkler et al., 2020).

The core components of ECM are collagen, proteoglycans, and glycoproteins. About 300 unique matrix macromolecules, including fibronectin, elastin, tenascin, and hyaluronic acid, constitute the core matrix of the ECM (Hynes and Naba, 2012). These ECM components are post-translationally modified by a series of secreted modifying enzymes, such as oxidases and proteases. A dynamically regulated physical scaffold built by these embellished components also exist dynamically biochemical and biomechanical properties of the ECM. Cell surface receptors (such as integrins, discoid domain receptors, and multimers) interact with ECM components, binding factors, sense the biochemical and mechanical properties of the ECM, mediate cell adhesion and cell signaling, thereby regulating proliferation, differentiation, migration and apoptosis (Hastings et al., 2019). In the physiological state, the production and degradation of ECM proteins and receptors maintain a homeostasis to ensure normal cell function. During the tumorigenic ECM remodeling, proteins and receptors undergo tumorigenic changes in the processes of ECM deposition (Figure 2A), ECM modification (Figure 2B) and ECM degradation (Figure 2C), which promote the growth, proliferation and metastasis of tumor cells (Winkler et al., 2020).

The abundance and composition of components are main variable during ECM deposition. Thus, the biochemical and mechanical properties are consist with these variation. ECM is produced mainly by CAFs, which are the main culprits in pathological changes such as fibrosis and canceration (Barbazan and Matic Vignjevic, 2019). CAFs are distinguished by α-smooth muscle actin (α-SMA) expression (Chen and Song, 2019). Various pro-fibrotic growth factors and inflammatory factors have an effect on the activation and differentiation of myofibroblast phenotype, such as TGF-α, TGF-β, fibroblast growth factor (FGF-2), platelet-derived growth factor (PDGF), epidermal growth factor (EGF) (Heneberg, 2016). Especially through active TGF-β signaling, CAFs deposit abundant collagen and express cytokines, promotes the recruitment and activation of immune cells, thereby establishing an oncogenic and pro-inflammatory cancer niche (Elyada et al., 2019). The deposition of cells inevitably lead to increase in ECM composition, including collagen, ECM modifying enzymes (such as lysyl oxidase (LOX) and LOX-like protein), fibronectin, hyaluronic acid, tenascin C and laminin. Deposition of fibrillar collagen is the most common tumorigenic change in the ECM and can directly promote tumor growth (Fang et al., 2014; Zhou et al., 2017). Deposition of fibronectin, hyaluronic acid and tenascin C lead to fibrotic phenotype (hyperplasia of connective tissue), a key feature of various cancers (Iacobuzio-Donahue et al., 2002). While laminin is involved in angiogenesis, especially in the process of vascular maturation. Deposition of ECM-modifying proteins hyaluronan and proteoglycan-linked protein 1 (HAPLN1) affects the integrity of the lymphatic vasculature, resulting in increased metastasis. Low-molecular-mass HA oligosaccharides (LMM-HA) have been linked with poor prognosis in several tumors (Lipponen et al., 2001; Schmaus et al., 2014). Dysregulated HA synthase and HA-degrading hyaluronidase lead to elevated LMM-HA, which interacts with the cell surface receptor CD44 to modulate pro-tumor signaling cascades (including glycolysis) (Sullivan et al., 2018).

Tumorigenic ECM modification including chemical modification at the post-translational level and force-mediated ECM remodeling (Wolf and Friedl, 2011; Nguyen-Ngoc et al., 2012). Chemical modification alters the biochemical properties and structural characteristics of the ECM(Karsdal et al., 2013). The main proteins involved are LOX, LOXLs, and tissue transglutaminase 2 (TG2) (Yuzhalin et al., 2018). Force-mediated ECM remodeling affects ECM tissue by aligning ECM fibers and opening cell migration channels. The main mechanical sensors involved are integrins, Rho kinase (ROCK), Disc domain receptors (DDR), Piezo1, YAP/TAZ (Jiang et al., 2022). In normal soft tissue, collagen fibers in the interstitial stroma that coil and run parallel to the epithelial layer may prevent cancer development by activating tumor suppressor phenotypes (Maller et al., 2013). However, collagen fibers near the tumor boundary are linear and vertically oriented, supporting the invasive growth of the tumor (Levental et al., 2009; Conklin et al., 2011). Overexpression of LOX and LOXLs increases fibrosis and ECM stiffness, and promotes tumorigenesis and metastasis (Barker et al., 2012). Overexpression of TG2 in cancer cells increases ECM cross-linking, affecting mechanical properties and cell-matrix signaling (Levental et al., 2009). Fibronectin glycosylation enhances the invasiveness of urothelial cancer cells (Richter et al., 2008). Increased glycosylation of transmembrane proteins results to bulky glycocalyx, applying tension on ECM-bound integrins which are important receptors for ECM components (Paszek et al., 2014). During this modification, mechanical forces exerted by aggregated integrins would result in nonproteolytic destruction of the basement membrane, allowing invasion of cancer cells (Kechagia et al., 2019).

CAFs, cancer cells and recruited bone marrow-derived cells secrete proteases that cleave and degrade the ECM: such as MMPs, disintegrins and metalloproteinases (ADAMs) (Lu et al., 2011). MMPs are involved in the physiological and pathological degradation of collagen (Mahalanobish et al., 2020). MMP-1, MMP-8, MMP-13 and MMP-14 cleave fibril-forming collagens I, II and III, while MMP-2 and MMP-9 cleave denatured collagen and collagen IV(Kessenbrock et al., 2010). ECM degradation is an important driver of cancer cell motility (Kai et al., 2019). The binding of soluble signaling molecules such as growth factors (GFs) to the ECM renders them insoluble and inactive, while proteases enables their release (Martino and Hubbell, 2010). The activity of proteases is counteracted by protease inhibitors (e.g., TIMP, a tissue inhibitor of metalloproteinases) (Saw et al., 2019). After ECM degradation, proteolytic fragment release matrix-bound matrikines (Mott and Werb, 2004; Wells et al., 2015). These factors induce pro-tumor ECM signaling that promotes tumor proliferation, migration, invasion, and angiogenesis (Ricard-Blum and Salza, 2014; Papadas et al., 2020). Matrikines also induce the expression and activation of MMPs, including membrane type-1 matrix metalloproteinase (MT1-MMP) and MMP-2 (Brassart et al., 1998). These combined changes in ECM make a hypoxic environment exist for cancer cells. Neutrophils secrete potent MMP-9, which degrades ECM and releases matrix-bound VEGF, creating a concentration gradient of new angiogenic sprouts (Ardi et al., 2007). Finally, upon stimulation of dense ECM, tumor cells may acquire endothelial-like functions and mimic the vasculature connected to blood vessels.

Overall, changes in the abundance of ECM components lead to different tissue densities and stiffnesses, ECM degradation leads to the release of GFs and cytokines secluded by ECM, which induce tumor cell growth, angiogenesis, and inflammation (Kai et al., 2019). Furthermore, the ECM interacts with neighboring cells and initiates multiple cellular signaling cascades. ECM deposition, modification, and degradation are important mechanisms that dynamically regulate ECM abundance and structure. The ECM remodeling trilogy is present simultaneously during tumorigenesis, but with different emphasis at different stages. Therefore, finding drugs to normalize ECM abundance and structure in the early, middle and late stages of cancer therapy is the research goal in the sequential treatment of cancer.

## 3 Natural products normalize ECM

### 3.1 Flavonoids

#### 3.1.1 Reduce ECM deposition

Flavonoids reduce ECM deposition mainly by reducing ECM components such as hyaluronic acid, laminin, fibronectin, etc., They work by inhibiting the NF-kB, JAK2/STAT1/2, p38 MAPK, JNK and TGF-β signaling pathways, of which TGF-β signaling is the most critical target. Ampelopsin reduces ECM deposition by lessening the amount of collage I, α-SMA, TIMPs, p-Smad3, TGF-β1 and increasing the expression of MMP-9 (Ma et al., 2019). Pectolinarigenin, reported as a STAT3 inhibitor, blocks the phosphorylation of Smad3 and STAT3, and downregulates the major fibrotic gene and protein expression of TGF-β, α-SMA, COL-1, and fibronectin in TGF-β1-stimulated fibroblasts (Li et al., 2021b). Neohesperidin not only decreases the TGF-β1-induced myofibroblast differentiation and ECM production, but also inhibit fibroblast migration (Guo et al., 2019). Curcumin potently reduces levels of hyaluronic acid, laminin, fibronectin, α1 (1) collagen, procollagen III, α-SMA (Zhang et al., 2012). Further research revealed that the effect is associated with modulation of CBRs system (Zhang et al., 2013). Similarly, ginkgetin reduces the expression of collagen IV, fibronectin, and laminin via mediating AMPK/mTOR axis (Wei et al., 2021). Eupatilin increases the expressions of α-SMA, whereas expressions of ECM proteins type I collagen and fibronectin are reduced (Li et al., 2020b). Isoliquiritigenin suppresses inflammation cytokines, excessive deposition of ECM and oxidative stress-induced apoptosis via nuclear factor-erythroid 2 related factor 2 (Nrf2) and NF-kB pathways (Xiong et al., 2018). Morin inhibits ECM generation through restraining the activation of p38 MAPK and JNK signaling pathways (Ke et al., 2016).

Some flavonoids simultaneously reduce ECM deposition and inhibit ECM degradation. For example, apigenin is shown to reduce the ECM deposition, but also possess the potential of preserving collagen matrix from breakdown. Apigenin inhibit ECM production in TGF-β1-stimulated CFs, and the mechanisms partly ascribed to the reduction of miR-155-5p expression and subsequent increment of c-Ski expression, which lead to the inhibition of Smad2/3 and p-Smad2/3 (Wang et al., 2021). Furthermore, apigenin and wogonin primarily lessen ECM degradation by blocking c-Fos/activator protein-1 (AP-1) and JAK2/STAT1/2 pathways (Lim et al., 2011).

#### 3.1.2 Alter ECM modification

In the force-mediated ECM remodeling, integrins play an important role by binding to the ECM molecules and applying mechanical force to ECM molecules and cancer cell. Silibinin interferes with the interaction between prostate cancer cells and fibronectin, thereby inhibiting their motility, invasiveness and survival. Studies have shown that silibinin modulates fibronectin induced expression of integrins (α5, αV, β1, and β3), actin remodeling (e.g., local adhesion kinase), apoptosis, EMT, and signaling molecules related to cell survival (Deep et al., 2014). Changes in surface integrins affect cell migration, proliferation, and ECM production. Quercetin alter the interaction between fibroblasts and ECM by up-regulating αV integrin and down-regulating β1 integrin on fibroblasts (Doersch and Newell-Rogers, 2017). Similarly, baicalein may be used to treat breast cancer by interfering with the interaction of ECM with cancer cells. Fibronectin-induced migration, invasion and F-actin remodeling are significantly inhibited by baicalein (Chen et al., 2019b).

#### 3.1.3 Inhibit degradation of ECM

MMPs are important accessory molecules in the process of tumor cell metastasis, which can cause excessive ECM degradation and cancer cell invasion. MMPS are also considered as promising targets for tumor therapy. Most flavonoids are potential candidates for MMPs inhibitors. MMP-2 and MMP-9 degrade ECM to release essential components of neovascularization. Silibinin, naringin and naringenin can inhibit MMP2/9 to reduce angiogenesis. Silibinin inhibits the expression of urokinase-type plasminogen activator (u-PA) and MMP-2 in human osteosarcoma MG-63 cells by down-regulating the induction of adhesion spot kinase and ERK-dependent c-jun/activator protein-1 (AP-1) (Hsieh et al., 2007). Simultaneously, silibinin significantly lessens the expression and activity of MMP-9 promoted by EGFR signaling (Liang et al., 2012). Naringin attenuates MAPK signaling pathways including p38, JNK and ERK, leading to downregulation of MMP-2/9 (Aroui et al., 2016). The main effect of naringenin is to inhibit pERK1/2, TLR and TGF-β pathways, which significantly reduces ECM synthesis and deposition. Other effects include inhibition of VEGF, induction of apoptosis and regulation of MAPK pathway. (Jung et al., 2013; Hernandez-Aquino and Muriel, 2018).

Oroxylin A also inhibits MMP-2/9 in breast cancer. Further elucidation of the mechanism reveals that it increases the expression of TIMP-2 and represses the PMA-induced translocation of PKC δ, phosphorylation of ERK1/2 and binding activity of the transcription factor AP-1 which are upstream signaling molecules in MMP-2/9 expression (Sun et al., 2009; Lu et al., 2012). Similarly, kaempferol inhibits MMP-2 and TIMP-2 transcription by suppressing c-Jun activity and phosphorylation of ERK1/2. It also reduces human breast carcinoma cells invasion through blocking the PKC δ/MAPK/AP-1 cascade and subsequent MMP-9 (Lin et al., 2013; Li et al., 2015b). In addition, kaempferol can inhibit other matrix degrading enzymes (MMP-1, MMP-3, MMP-13, disintegrin, etc.), consequently abolishes the degradation of collagen II (Huang et al., 2018). Wogonin exerts its anticancer effects in breast cancer may through targeting 5-LO/BLT2/ERK/IL-8/MMP-9 signaling cascade. By inhibiting the BLT2/ERK-linked cascade, wogonin attenuates the expression of IL-8 and MMP-9 (Chen et al., 2011; Go et al., 2018). Furthermore, wogonin causes suppression of melanoma cell B16-F10 migration, adhesion, invasion and actin remodeling by inhibiting MMP-2 (Zhao et al., 2014). Orientin suppresses MMP-9 expression through inhibiting TPA-induced PKC α and ERK activation, as well as the nuclear translocation of AP-1 and STAT3 (Kim et al., 2018). In the glioblastoma multiforme (GBM), tricetin reduces MMP-2 production at transcriptional level. Specificity protein-1 (SP-1) is an promising object of tricetin for inhibiting MMP-2-mediated cell motility. Preventing the ERK pathway would inhibit the effect, and further enhances the anti-invasive ability of tricetin against GBM cells. The combination of tricetin and an ERK inhibitor may be a good strategy for preventing GBM invasion (Chao et al., 2015). In the A549 cell line, fisetin induces apoptosis by decreasing the expression of MMP-2/9, increases the expression of E-cadherin via targeting the ERK signaling pathway (Wang and Huang, 2018). In colorectal cancer, baicalein suppresses cell invasion via inhibiting the ERK signaling pathways and the expression of the MMP-2/-9 (Chai et al., 2017). Chrysin and daidzein perform their antitumor effect in part due to the downregulation of CXCL-1, ERK, AKT, and MMP-9 (Salama and Allam, 2021).

TGF-β/Smad pathway occupies an important position in ECM remodeling. Regarded as a TGF-β inhibitor, Hesperetin works on the TGF-β1/Smad pathway-mediated ECM progression and restores the upregulation of IL-1β, PTGS2, and MMP-13 induced by TNF-α, reverses the degradation of the ECM, therefore it has promising therapeutic effects on various cancers (Kong et al., 2018; Wu et al., 2021). Curcumin significantly suppresses the expression of TGF-β1 and phospho-smad2/3, upregulates smad7, increases the amount of α-SMA expressing myofibroblasts, inhibits activity of MMPs, preserves ECM from degradation and also attenuates collagen deposition (Wang et al., 2012). It inhibits NO, PGE2, IL-6, IL-8, and MMP-3 production in a concentration-dependent manner (Mathy-Hartert et al., 2009). In addition, some genes relating to ECM-receptor interaction pathway, such as GP1BB, COL9A3, and AGRN, could be inhibited by curcumin (Mao et al., 2021).

Quercetin inhibits the degradation of ECM via up-regulating SOD and TIMP-1, down-regulating MMP-13 and increasing the expression of collagen II (Wei et al., 2019; Guo et al., 2021). Noteworthy, quercetin also induces activation of MMP-2/9 (Hernandez-Ortega et al., 2012). Isoliquiritigenin decreases expression level of collagen X (Col X), MMP-13 and exerts anti-angiogenesis effects through direct suppression of MMP-2 (Ji et al., 2018). Glabridin upregulates the expression levels of ECM related genes, such as collagen II, proteoglycan 4, SRY-box 9 and aggrecan (Dai et al., 2021). The addition of genistein results in downregulation of the transcription of most of MMPs genes in MDA-MB-231 and MCF-7 cells (Kousidou et al., 2005). Isorhamnetin obviously blocks MDA-MB-231 cell invasion via downregulating MMP-2/9, which is potentially associated with the inhibition of p38 MAPK and STAT3 (Li et al., 2015a). At the same time, Isorhamnetin could reduce mRNA expression of TNF-α, IL-1β, IL-6, IL-12, and MMP-1 (Jnawali et al., 2016). Ononin inhibits the expression of MMP-13 and alleviates the decomposition of collagen II protein by downregulating the MAPK and NF-κB signaling pathways (Xu et al., 2022). Nobiletin suppresses MMP-9 expression through modulating p38 MAPK without cytotoxicity (Kim. et al., 2014).

These findings constitute the molecular basis for the antiinvasive and anti-metastatic effects of flavonoids, and shed light on the investigation of flavonoids on cancer metastasis. Therefore, we proposes that flavonoids might be considered as a therapeutic potential candidate for the treatment of cancer metastasis.

#### 3.1.4 Targeting CAFs

A study found that curcumin effectively inhibits TGF-β1-induced fibroblasts differentiation through Smad-2 and p38 signaling pathways (Liu et al., 2016). Further research revealed that curcumin induces the apoptosis and cell cycle arrest of prostate-CAFs. The upregulation of ROS caused by curcumin triggers endoplasmic reticulum stress of CAFs via the PERK-eIF2 α-ATF4 axis (Zeng et al., 2020). Moreover, curcumin abrogates CAF-induced invasion and EMT, and lessens ROS production and CXCR4 and IL-6 receptor via blocking MAOA/mTOR/HIF-1α axis, thereby supporting the therapeutic effect of curcumin in prostate cancer (Du et al., 2015). Meanwhile, curcumin eliminate CAF’s ability of promoting invasion by increasing E-cadherin level and lowering vimentin level. In prostatic cancer, the observed increase in α-SMA and CAF-like phenotype is TGF-β2 dependent, which is strongly suppressed by silibinin. The finding emphasizes the potential application of silibinin in prostatic cancer prevention by targeting the CAF phenotype (Ting et al., 2015). Moreover, silibinin reduces MCP-1 and CAFs’ biomarkers (fibroblast activation protein, a-SMA, TGF-β2, vimentin, etc.), and significantly modulates the recruitment of immune cells in TME, inhibiting invasiveness in prostatic cancer (Ting et al., 2016).

### 3.2 Phenols

#### 3.2.1 Reduce ECM deposition

Most Phenols reduces ECM-related protein expression via NF-kB signaling or TGF-β-induced cellular responses, demonstrating their potential to reduce ECM deposition. Noteworthy, some phenols exert dual-directional regulation on ECM remolding. Resveratrol decreases the mRNA levels of fibronectin and the protein expression of collagen I and α-SMA, as well as β-catenin (Chen et al., 2019a). It also inhibits the generation of VEGF, MMP-3, MMP-9 and COX-2 to counteract IL-1β-mediated degradation. These gene are regulated by NF-kB. Nuclear translocation of the p65 subunit of NF-kB and its phosphorylation are consist with IL-1β-induced proteasome function and the dissociation of IkBa which suppressed by Resveratrol. (Shakibaei et al., 2008; Frischholz et al., 2020). The production of TNF-a, IL-6, fibronectin and collagen IV is inhibited by daphnetin. In addition, daphnetin enhances the expression of Nrf2 and inhibits the levels of p-Akt and p-p65. Thus, daphnetin inhibits ECM accumulation, oxidative stress, and inflammatory response. The effect is partially intermediated by Nrf2/keap1 and Akt/NF-kB pathways (Xu et al., 2019).

Garcimultiflorone K inhibited Smad protein phosphorylation, cell migration, and ECM expression induced by TGF-β. Mechanistic studies demonstrated that garcimultiflorone K suppresses TGF-β signaling by downregulating TGF-β receptor II (Huang et al., 2021b). Histological studies revealed that sesamin reduces CAFs activation and collagen accumulation in the ECM. Sesamin inhibits TGF-β1-stimulated expression of α-SMA, the accumulation of α-SMA-positive cells and expression of collagen I, as well as TGF-β1-induced Smad3 phosphorylation (Lin et al., 2014). Osthole significantly mitigates fibronectin, collagen IV, and laminin increased by TGF-β2 stimulation, suggesting it’s capable of reducing ECM expression (Fan et al., 2020).

#### 3.2.2 Alter ECM modification

Integrin-mediated ECM modification is crucial during the cell adhesion processes involved in carcinogenesis. Epigalloccatechin-3-gallate (EGCG) specifically antagonizes the migration of DAOY cells on collagen by up-regulating the expression of specific genes and proteins of the β-1 integrin subunit and improving the adhesion of cells (Pilorget et al., 2003). [10]-gingerol downregulates the expression of EGFR and β1-integrin, reverting the malignant phenotype of the breast cancer cells (Fuzer et al., 2017).

#### 3.2.3 Inhibit degradation of ECM

In HTB94 chondrosarcoma cells and breast cancer cells, resveratrol significantly suppresses the expression of Cyclin D1, c-Myc, SOX-2, MMP-2/9, and inhibits MMP-induced differentiation via the p38 kinase and JNK pathways as well as activation of Akt and STAT3. At the same time, resveratrol induces the expression of collagen II, SOX-9 and the production of sulfated proteoglycans (Gweon and Kim, 2014; Suh et al., 2018). Some structural analogues of natural products may have better pharmacological effects. For instance, 4,4′-dihydroxy-trans-stilbene (DHS), a synthetic analog of resveratrol, exerts a strong reduction in MMP-2/9 activities, concomitantly with a pronounced inhibition of cell adhesion, migration and invasion to ECM components. Meanwhile, modulation of E-cadherin is also observed in DHS-treated cells. These outcomes suggested that the two 4,4′-hydroxyl groups bound to the stilbenic backbone make DHS a more active molecule than resveratrol (Maccario et al., 2012).

Phenolics extracted from tea include Epicatechin-3-gallate (ECG), EGCG and catechins. ECG suppresses the invasion of highly metastatic A549 cells by reducing the activities of MMP-2 and uPA, simultaneously inhibits fibronectin and p-FAK (Huang et al., 2016). Catechin significantly decreases circulatory MMP-9 levels at a dose of 50 mg/kg (Addepalli and Suryavanshi, 2018). Previous studies have shown that EGCG inhibit prostate CAFs differentiation. ECM contraction is an enhancer of tumor cell invasion. Further researches showed that EGCG and luteolin inhibit TGF-β-induced ECM contraction and the downstream signaling of ERK and AKT. Furthermore, both of them reduce Rho A activation which is necessary for fibronectin expression. Thus, combined clinical use of these compounds could prevent or reverse cancer progression by targeting the TME. (Gray et al., 2014).

MMPs and VEGF exert critical effects during the ECM remodeling and angiogenesis. EGCG protect ARPE-19 cells against apoptosis and attenuate mRNA and protein expressions of MMP-9, VEGF, VEGFR-2 by inhibiting production of ROS(Lee et al., 2014). Procyanidins crosslink vascular ECM proteins, protect them against proteolysis by MMPs (Zhai et al., 2011). Erianthridin has shown obviously antimetastatic activity against non-small-cell lung cancer through Akt/mTOR/p70S6K-induced actin reorganization and MMP-2/9 expression (Pothongsrisit et al., 2021). Treatment of cultured B-cell lymphocytic leukemia patients’ cells with hyperforin results in an obvious inhibition of their capacity to secrete MMP-9. The hyperforin acts by reducing the production of the latent 92 kDa pro-enzyme and decreasing VEGF released by the leukemic cells (Quiney et al., 2006). Ellagic acid and rosmarinic acid both downregulate the MMP-13 and upregulate the collagen of type II, aggrecan and sulfated-proteoglycan synthesis. Ellagic acid acts via the NF-κB signaling, while the rosmarinic acid via ERK-1/2 and p38 kinase signaling pathways (Eo and Kim, 2017; Lin et al., 2020). Esculetin and fraxetin are both dihydroxycoumarins. Esculetin’s antiangiogenic action in LM8 cells have been due to the inhibition of MMP-2, TGF-β1 and VEGF productions at tumor sites (Kimura and Sumiyoshi, 2015). The coumarin derivative 4-methylesculetin (4-ME) is known to inhibit the elevation of hyaluronidase, MMP-13, MMP-3 and MMP-9 levels that cause ECM degeneration (Hemshekhar et al., 2013). Magnolol has been recognized as an antitumor reagent. In HCC bearing animal model, expression of phospho-ERK, NF-κBp65, MMP-9, VEGF, and CyclinD1 are significantly decreased by it. In addition, caspase-8 and caspase-9, the major extrinsic and intrinsic apoptosis signaling factors, are both enhanced by magnolol (Tsai et al., 2020).

Zingerone is a phenolic alkanone with anti-inflammatory and antioxidant effects. Acetyl zingerone is a structural analogue of Zingerone. Both of them upregulate Notch pathway gene expression and downregulate expression of genes linked to ECM disassembly, thereby improving ECM integrity. After structural improvement, Acetyl zingerone possesses a greater activity of suppressing MMP-3 and MMP-12 (Swindell et al., 2020).

#### 3.2.4 Targeting CAFs

Resveratrol can revoke the generation of IL-6 by CAFs. CAFs strongly induce IL-6-mediated cell motility in conditioned medium, but pretreatment with resveratrol completely prevented cancer cell motility, simultaneously reversing the N-to-E cadherin switch (Thongchot et al., 2018). Eugenol and decitabine can inhibit the expression of DNA methyltransferase genes DNMT1 and DNMT3A in breast CAF cells, and suppress the invasion/migration and proliferation potential of CAF cells by down-regulating E2F1, as well as their paracrine carcinogenic effects. The difference is that eugenol has a persistent inhibitory effect, whereas the effect of decitabine is transient. (Al-Kharashi et al., 2021).

### 3.3 Polysaccharides

Ascophyllan inhibits the migration and adhesion of B16 melanoma cells through lessening the expression of N-cadherin and enhancing the E-cadherin. Furthermore, ascophyllan inhibited the expression of MMP-9 mRNA and the secretion of MMP-9 protein, a process that may involve the ERK signaling pathway (Abu et al., 2015). Prunella vulgaris Polysaccharide could exert an antineoplastic effect on CAFs by inhibiting basic fibroblast growth factor (b-FGF) expression, thus inhibiting the growth of breast cancer cells indirectly (Hao et al., 2020).

### 3.4 Saponins

Polyphyllin II may be a potential antineoplastic drugs used in the prospective application by improving ECM. It arouses programmed cell death and suppresses metastases in murine lung adenocarcinoma, efficaciously attenuates hepatotoxic and inhibits pulmonary adenoma through down-regulating expression of MMP-9 and upregulating level of TIMP-2 (Man et al., 2015). Astragaloside Ⅳ can strongly inhibit the proliferation/migration/invasion-promoting capacities of gastric CAFs. It significantly upregulates microRNA-214 expression and downregulates microRNA-301a expression in gastric CAFs, reestablishes the microRNA expression balance subsequently elevates TIMP2 production and secretion, and suppresses macrophage colony-stimulating factors production and secretion (Wang et al., 2017b).

### 3.5 Terpenoids

Terpenoids regulate the ECM remodeling by decreasing the production of MMPs and reducing collagen deposition. Heparan sulfates are long, unbranched, negatively charged polysaccharides that are bound to core proteins. Heparanase cleaves the ECM by degrading heparan sulfate, which eventually leads to cell invasion and metastasis. In the melanoma, hinokitiol could suppress the expression of heparanase through lowering the phosphorylation of protein kinase B (Akt) and ERK, inhibiting the expression and activity of MMP-2/9 simultaneously (Huang et al., 2015; Wu et al., 2020). Artemisinin significantly inhibits the induction of extracellular matrix metalloproteinase inducer (EMMPRIN) and MMP-9 at both the transcriptional and translational levels via suppressing the PKCd/ERK/p38 cascade (Wang et al., 2011). Andrographolide inhibits MMP-2-mediated cell motility through blocking the ERK 1/2 pathway in GBM cells, as well as targeting the CREB. A combination of andrographolide and an ERK inhibitor might be a good strategy for preventing GBM metastasis (Yang et al., 2017). Asiatic acid could inhibit TGF-β1-induced expression of collagen I, suppress the phosphorylation of Smad2/3 and the expression of plasminogen activator inhibitor-1(PAI-1), while elevate Smad7 protein level (Bian et al., 2013).

### 3.6 Alkaloids

Most alkaloids inhibit MMP-2/9 through P38, PI3K/Akt and protein kinase C-a (PKCa)/ERK/nuclear factor kappa B (NF-jB) pathways respectively. The anticancer activity of α-Chaconine mainly includes inhibition of tumor cell proliferation, migration, invasion and induction of cell apoptosis. Researches reveals a new therapeutic potential for α-chaconine and α-solanine on anti-angiogenic therapy. They suppresses migration, invasion and tube formation of cancer cell through inhibiting MMP-2 activities, as well as JNK, PI3K/Akt and NF-kB signaling pathways (Lu et al., 2010a), (Lu et al., 2010b). Some reports demonstrated that α-tomatine prevent the invasion/migration of MCF-7 cells through blocking PKCa/ERK/NF-jB signaling pathways. Specifically, α-tomatine could inhibit the activation of ERK1/2 and PKCa involved in the downregulation of the enzyme activities and messenger RNA levels of MMP-2/9 induced by TPA. Next, α-tomatine also strongly inhibits TPA induced the activation of NF-jB and phospho-inhibitor of kappa Ba (Shi et al., 2013; Yelken et al., 2017). Aristolochic acid inhibits MMP-9 through the NF-jB signal pathway, implying it may be involved in alteration of matrix homeostasis (Wu et al., 2011).

Overproduction of MMPs and EMMPRIN by monocytes/macrophages leads to the degradation of ECM. IL-1β prompts the expression of MMPs and IL-6 through toll-like receptor 4 (TLR4)/NF-jB axis, while oxymatrine reduce the expression of MMPs and TNF-a induced by IL-1β(Wei et al., 2020). Berberine significantly inhibits IGF-1R expression and decreases MMP-2/9, a-SMA, but also reduces collagen I expression (Li et al., 2018a). Further research showed that berberine lessens MMP-9 and EMMPRIN generation through inhibiting the activation of p38 pathway in PMA-induced macrophages (Huang et al., 2011). Solasodine was found to inhibit proliferation in various tumor cells. It reduces the mRNA level of MMP-2/9 and EMMPRIN, increases the production of TIMP-1/2, and simultaneously suppresses PI3K and Akt phosphorylation (Shen et al., 2017). Evodiamine inhibits cell migration and invasion abilities by down-regulating expression of MMP-9, uPA and uPAR. Evodiamine-induced G0/G1 arrest and apoptosis are linked with a lessening in Bcl-2, cyclin D1 and cyclin-dependent kinase 6 (CDK6) expression and an increment in Bax and p27Kip1 expression. Furthermore, it also can regulates p-ERK and p-p38 MAPK expression (Du et al., 2013).

### 3.7 Quinones

Thymoquinone, a major active compound derived from the medicinal Nigella sativa, may reduce ECM accumulation by enhancing the phosphorylation AMPK. Thymoquinone significantly reduces protein and mRNA expression of α-SMA, collagen and TIMP-1, accompanied by downregulating the expression of TLR4 and proinflammatory cytokine levels, also inhibits PI3K phosphorylation (Bai et al., 2014). Analogously, shikonin significantly inhibits ECM formation by downregulating the TGF-β1 expression and maintaining the normal equilibrium between MMP-2 and TIMP-1 (Liu et al., 2019). Cross-linking formation play a critical role in ECM formation and homeostasis. Emodin was found to be a strong enhancer of LOXL1 expression that promotes ECM cross-linking formation by using the ZsGreen reporter system for drug screening, suggesting that emodin may be an effective drug or complement to ECM homeostasis (Jian et al., 2014).

## 4 Natural products affect the TAM-ECM-cancer cell crosstalk

### 4.1 Flavonoids

TAMs occupy an important position in modulating the TME and favoring cancer metastases. TAMs-targeted therapy is a promising approach that could be used to reverse the immunosuppressive tumor microenvironment. In osteosarcoma LM8-bearing mice, wogonin inhibits tumor growth and metastasis to multiple organs, angiogenesis, lymphangiogenesis and reduces TAM numbers. The effects are associated with the reduction in VEGF-C-induced VEGFR-3 phosphorylation by the inhibition of COX-2 expression and IL-1β production in TAMs (Kimura and Sumiyoshi, 2013). The therapeutic effect of silibinin in inhibiting lung tumor growth and regression through an antiangiogenic mechanism appears to be mediated by reduction of TAM and cytokines, inhibition of HIF-1α, NF-κB and STAT-3 activation, and upregulation of angiogenic inhibitors Ang-2 and Tie-2 (Tyagi et al., 2009). In glioblastomas, Isoliquiritigenin decreasing secretion of 20-HETE, VEGF and TGF-βreduces, accompanied with the reduction of tumor burden and normalization of vasculature. Furthermore, isoliquiritigenin intervenes crosstalk between TAMs and endothelial progenitor cells during angiogenesis (Wang et al., 2017a). TAMs achieves immunosuppression by secreting immunosuppressive molecules such as IL-10, TGF-β. Luteolin has been demonstrated that it exerted significant tumor suppressive activity on several cancers, by suppressing the secretion of IL-6, IL-12 and TNF-α in macrophages (Chen et al., 2018). Kumatakenin shows antineoplastic activities by eliciting apoptosis of ovarian cancer cells and suppressing the alternative activation of TAM. It inhibits the generation of M2 markers, IL-10, MMP-2/9, VEGF, and MCP-1, which is major determinants of macrophage recruitment at tumor sites (Woo et al., 2017).

Curcumin exhibits a potent anti-inflammatory activity which partly mediated by inhibiting the JNK signaling pathway. It is worth emphasizing the anti-inflammatory effects of curcumin in macrophages and the role of altering the macrophage M1/M2 equilibrium in TME. Mechanism of curcumin to immune modulation might be associated with its ability to enhance IL-10-mediated effects (Shiri et al., 2015; Somchit. et al., 2018). Baohuoside I is the key bioactive compound of XIAOPI formula,a newly approved drug by the State Food and Drug Administration of China. Mechanistic investigations demonstrated TAMs/CXCL1 is the crucial target of BHS in suppressing breast cancer metastasis. Baohuoside I can inhibit the M2 phenotype polarization of TAMs, thereby attenuating the expression and secretion of CXCL1 (Wang et al., 2020). Icariin impedes the development of pancreatic cancer in mice by regulating the tumor immune microenvironment. Icariin suppresses the polarization of RAW 264.7 cells into M2 macrophages by down-regulating ARG1 and MRC1 expression and blocking the IL4-STAT6 signaling pathway (Zheng et al., 2019).

### 4.2 Phenols

CD4+Foxp3+ regulatory T cells (Tregs) in the TME restrict antitumor immunity, contributing to tumor aggression and poor survival. CD8+CD122+ Tregs are more potent in immunosuppression than CD4+Foxp3+ Tregs. Studies have validated that resveratrol exerts its antineoplastic effects by suppressing CD4+Foxp3+ and M2-like macrophages. In the recent study, resveratrol inhibits the tumor growth in hepatocellular carcinoma model by decreasing the frequency of CD8+CD122+ Tregs. Furthermore, immunosuppressive cytokines such as TGF-β1 and IL-10 in tumor tissues were decreased by resveratrol, whereas antitumor cytokines TNF-α and IFN-γ were increased. Resveratrol also inhibits the activation of STAT3 signaling and reduces the percentage of M2-like macrophages (Zhang et al., 2020). Locally aggregated IFN-γ at the tumor site regulate TAM status. Under IFN-γ exposure, TAMs change their phenotypes to the M1-like morphology and intracellular granular pattern, accompanied with a reduction level of immunosuppressive and tumor progressive mediators. Synthetic resveratrol analog HS-1793 significantly increases IFN-γ secreting cells in splenocytes and decreases CD206^+^ macrophage infiltration (Jeong et al., 2014). Another study found that resveratrol significantly inhibited the tumor growth associated with decreased expression of p-STAT3 (Kimura and Sumiyoshi, 2016; Zhao et al., 2018).

The interaction between TAM and cancer cells provides a perspective of future therapeutic approaches. EGCG exerts anticancer activity by regulating of TAMs in TME. The expression levels of monocyte chemokines (csf-1 and ccl-2) in tumor cells of mice treated with EGCG were lower, and the cytokine was skewed from M2-to M1, which was manifested as the decrease of IL-6 and TGF-β, and the increase of TNF-α. EGCG upregulates miR-16 in tumor cells, enabling miR-16 translocate to TAM via exosomes and inhibit TAM invasion and M2 polarization (Jang. et al., 2013). Gallic acid and Caffeic acid block the processes of angiogenesis and tumor growth via protection of the tumoricidal efficacy of M1 macrophages and inhibition of proangiogenic factors, particularly VEGF, MMP-2/9, and cyclooxygenase-2 (COX-2) activity (Orsolic et al., 2020). Esculetin and fraxetin reduces the p-STAT3 and inhibits the production of IL-10, MCP-1 and TGF-β1 during the differentiation of M2 macrophages, thereby regulating the activation of TAM(Kimura and Sumiyoshi, 2015).

### 4.3 Polysaccharides

CMPB90-1, a novel natural polysaccharide from Cordyceps sinensis, activates p38, Akt and NF-κB by binding to TLR2, which resets TAMS from M2 phenotype to M1 phenotype. This process also involves the functional inhibition of the immune checkpoint programmed death ligand-1 (PD-L1)/programmed death 1 (PD-1) axis and T lymphocytes. These findings suggest that CMPB90-1 may be developed as a potential tumor immunotherapy reagent (Bi et al., 2020). Similarly, Astragalus polysaccharin inhibited the HCC-like phenotype in a mouse hepatocellular carcinoma model by inhibiting the M2 polarization of TAM. It enhanced the expression of M1 macrophage markers and the proportion of M1 macrophages, but the opposite effect was observed for M2 macrophages (Li et al., 2021a). It was shown that oral administration of granulosa yeast-derived β-Glucan significantly delayed cancer progress, which was linked with TAM phenotypic (Liu et al., 2015). These findings establish a new paradigm for macrophage polarization and immunosuppressive TAM conversion and shed light on the action mode of natural products treatment in cancer.

### 4.4 Saponins

Ginsenoside Rh2 is a promising anti-tumor monomer compound for lung cancer. *In vivo*, G-Rh2 can reduce the expression levels of M2 macrophage markers CD206 and VEGF, and contribute to the conversion of TAM from M2 subsets to M1, thereby preventing the migration of lung cancer cells. In addition, G-Rh2 can also reduce the expression levels of MMP-2 and MMP-9 (Li et al., 2018b). Dioscin is an herbal steroid saponin that promotes the secretion of proinflammatory cytokines (IL-6, TNF-α, and IL-1β) by promoting the M2 to M1 phenotypic transition (Kou et al., 2017).

### 4.5 Terpenoids

The inhibitory effect of terpenoids on TAM viability, differentiation, and cytokine production might elucidate the major mechanisms underlying its antitumor activity. The CCL2/CCR2 axis is an important mechanism of mediated macrophage infiltration. A natural product in fir, named 747, is structurally related to kaempferol and has sensitivity and selectivity similar to CCR2 antagonists. 747 blocked tumor-infiltrating macrophage-mediated immune suppression, increased the number of CD8+T cells in tumors, and inhibited *in situ* and subcutaneous tumor growth. In addition, 747 enhance the therapeutic effect of low-doses of sorafenib without significant toxicity. This study suggests that the combination of immunomodulators and chemotherapeutic agents may be a new way to treat tumors (Yao et al., 2017). Paeoniflorin (Wu et al., 2015), triptolide (Li et al., 2020a), corosolic acid (CA) and oleanolic acid (OA) can inhibit the differentiation of macrophages into M2 phenotype, suppress the expression of M2 markers and anti-inflammatory factors such as CD206, CD163, CD204, and IL-10. In addition, CA and OA also directly inhibit the proliferation of tumor cells and sensitize tumor cells to anti-cancer drugs such as doxorubicin and cisplatin (Fujiwara et al., 2011; Fujiwara et al., 2014).

### 4.6 Quinones

It has been shown that emodin delay tumor growth by inhibiting macrophage invasion and M2-like polarization, while increasing T-cell activation and reducing tumor angiogenesis. It is worth noting that emodin can interfere with the cancer-promoting feed-forward interaction between tumor cells and macrophages, thereby improving the immunosuppressive status of TME. Recent studies have found that emodin can block the TGF-β1-mediated crosstalk between TAMS and breast cancer cells, inhibit the epithelial cell transformation of breast cancer cells and the formation of cancer stem cells (Liu et al., 2020). Another study showed that emodin inhibited the secretion of MCP-1 and CSF-1 by tumor cells, thereby reducing macrophage migration to and adhesion to tumor cells. In conclusion, emodin inhibits tumor growth and metastasis by effectively blocking the feed-forward loop between breast cancer cells and macrophages (Iwanowycz et al., 2016). The specific dosage, other information and structural formula of all the natural products are shown in Tables 1, 2.

**TABLE 1:** The Table of Natural products targeting ECM.

1.Flavonoid				
Name	Models	Dosage range	Targets/pathway/process	Reference
Curcumin	Ketamine and zylaxine (rats)	150 mg/kg	MMPs, TGF-β1, phospho-Smad2/3, Smad7	100
	Second passage CFs	20 µmol/l	Smad-2, p38, TGF-β1	113
	Colorectal CSCs	1, 5, 25 μM	TFAP2A	102
	Human chondrocytes and cartilage explants	5-20 μM	NO, PGE2, IL-6, IL-8, MMP-3, ^35^S-GAG	101
	CCl_4_ (rats)	100, 200, 400 mg/kg	CBR1	71
	CCl_4_ (rats)	200 mg/kg	HA, laminin, procollagen III, fibronectin, α-SMA, α1(1) collagen	70
	PC3 cells	25 µM	MAOA/mTOR/HIF-1α, CXCR4, IL 6, ROS	115
	Prostate-CAFs, PC-3	0, 10, 20, 30 μM	ROS, PERK/eIF2α/ATF4, CAF	114
	BALB/c (rats)	40, 80 mg/kg	IL-10, IL-12, STAT3, STAT4, M2 macrophage	173
Silibinin	PC3 cells	50–200 µM	Integrins (α5, αV, β1, β3)	78
	Human RCC cell lines (ACHN, OS-RC-2 and SW839)	0, 25, 50, 75 µM	ERK1/2, EGFR/MMP-9	82
	MG-63 cells	0, 5, 10, 15, 20, 25, 30 µM	u-PA, MMP-2, ERK1/2, AP-1, c-Jun	81
	Urethane injected mice	742 mg/kg	Ang-2, Tie-2, TIMP-1, TIMP-2, HIF-1α, NF-κβ, STAT-3	168
	PC3 cells	30, 60, 90 µM	α-SMA, CAF	116
	RWPE-1, WPE-1 NA-22, WPE-1 NB-14 and PC3	90 µM	MCP-1, fibroblast activation protein, α-SMA, TGF-β2, vimentin	117
Kaempferol	Rat chondrocytes	0, 5, 10, 20 µM	IL-1β, MMP-1, MMP-3/13, disintegrin, p38	90
	MDA-MB-231,B16F10	10, 20, 40 µmol/L	PKCδ/MAPK/AP-1, MMP-9	88
	C57BL/6 mice	50,100, 200 mg/kg		
	SCC-4	0–100 μM	MMP-2, TIMP-2, ERK1/2, Jun	89
Quercetin	C57Bl/6J mice	10 μM	surface αV, β1 integrin	79
	OA rabbits	25 mg/kg	SOD, TIMP-1, MMP-13	104
	CCl4/mineral oil injected rats	100 mg/kg	MMP-2/9	106
	Rat chondrocytes	0, 12.5, 25, 50, 100, 200 μM	MMP-13, p110α/AKT/mTOR, collagen II gene	105
Isoliquiritigenin	HK-2 human renal proximal tubule cells	2.5, 5, 10 μM	Nrf2, NF-kB	74
	ACLT mice	10, 20, 40 mg/kg	Col X, MMP-13, MMP-2	107
	C6 glioma cells, U87 glioma cells	10, 20 mg/kg	20-HETE, VEGF, TGF-β, TAM	170
Isorhamnetin	INF-γ (MRC-5 Cells)	20 μM	TNF-α, I L-1β, IL-6, IL-12, MMP1	111
	MDA-MB-231	10, 20, 40 μM	MMP-2/9, p38 MAPK, STAT3	110
Wogonin	MDA-MB-231	15, 30, 60 μM	MMP-9, PKCδ, ERK1/2	91
	B16-F10	15, 30, 60 μM	MMP-2, Rac1	93
	B16-F10 (mice)	15-60 mg/kg		
	MDA-MB-231	20 µM	IL-8, MMP-9, BLT2 /ERK, 5-LO/BLT2/ERK/IL-8/MMP-9	92
	MDA-MB-231 (mice)	20 mg/kg		
	LM8 cells (mice)	25, 50 mg/kg	COX-2, IL-1β, VEGFR-3	168
	LM8 cells	0.1–100 µM		
	SW1353 cells	10, 25 μM	c-Fos/AP-1, JAK2/STAT1/2, MMP-13	77
Apigenin	CFs	6–24 μM	miR-155-5p, c-Ski, Smad2/3, p-Smad2/3	76
	SW1353 cells	5-25 μM	c-Fos/AP-1, JAK2/STAT1/2, MMP-13	77
Oroxylin A	MDA-MB-435	1, 10, 100 µM	MMP-2/9, ERK1/2	86
	MDA-MB-231	1, 4, 16 µM	TIMP-2, PMA, PKCδ, ERK1/2, AP-1	87
	B16-F10 (C57BL/6 mice)	20, 40, 80 mg/kg		
Hesperetin	Rat Chondrocytes	1, 5, 10, 20, 50, 100 μM	IL-1β, PTGS2, MMP-13	99
	OA rats	10 μM		
	BDL (mice)	200 mg/kg	TGF-β1/Smad	100
	HSC-T6 cell	0, 5, 10, 20, 50, 100 µM		
Baicalein	CRC HT29 and DLD1 cell	0–120 µM	ERK, MMP-2/9 mice	97
	DLD1 implanted Balb/c athymic nude	20 mg/kg		
	Mice	30 mg/kg	calpain-2	80
	MCF-10A	—		
Tricetin	GBM 8401, U87	0, 20, 40, 80 µM	MMP-2, SP-1	95
Chrysin	Colorectal cancer models (mice)	125, 250 mg/kg	MMP-9, CXCL1, AREG, ERK, AKT	98
	SW620	—		
Daidzein	Colorectal cancer models (mice)	5, 10 mg/kg	MMP-9, CXCL1, AREG, ERK, AKT	98
	SW620	—		
Glabridin	human OA chondrocytes	—	Collagen II, ACAN, SOX9, PRG4	108
	OA rats	100 μL		
Genistein	MCF-7, BT-20, MDA-MB-231, MCF-12A	35, 100 μM	MMPs	109
Morin	rat glomerular MCs	25, 50 μM	p38 MAPK, JNK	75
Icariin	pancreatic cancer models (mice)	120 mg/kg	ARG1mRNA, MRC1mRNA, IL4-STAT6	176
Kumatakenin	A2780, SKOV3	—	IL-10, MMP-2/9, VEGF	172
Luteolin	rats	20 mg/kg	IL-6, IL-12, TNF-α, NF-κB, M1 macrophage	171
Ononin	rat Chondrocytes	0–100 μM	MAPK, NF-κB, TNF-α, IL-6, MMP-13	112
Ampelopsin	CCl_4_ (mice)	125, 250 mg/kg	collage I, α-SMA, TIMP-1, TGF-β1, p-Smad3, MMP-9, SIRT1SIRT1/TGF-β1/Smad3	67
	mouse hepatic stellate cells	25, 50, 100 μM		
Eupatilin	Human ASMCs	0, 10, 20, 40, 80 μM	NF-κB, STAT3, AKT	73
Fisetin	A549	10, 40 µM	c-myc, cyclin-D1, COX-2, CXCR4, MMP-2/9, E-cadherin	96
Nobiletin	hDFs	—	p38 MAPK, MMP-9	113
Orientin	TPA (MCF-7)	50 nM	MMP-9, IL-8, PKCα, ERK, AP-1, STAT3	94
Pectolinarigenin	UUO mice	25 mg/kg	TGFβ, α-SMA, COL-1, FN, SMAD3, STAT3	68
	NRK-49F	50 μМ		
Neohesperidin	pulmonary fibrosis mice	20 mg/kg	TGF-β1/Smad3	69
	CAGA-NIH-3T3	—		
	MLg	20 µM		
Baohuoside i	MCF-10A, HBL100, BT549, Raw264.7	—	TAMs/CXCL1	175
Ginkgetin	HBZY- 1	2-80 μM	AMPK/mTOR	72
Naringin	U87	0, 5, 10, 15 μM	ERK, JNK, p38, MAPK, MMP-2, MMP-9	83
Naringenin	fibroblasts	0–160 μM	α-SMA, pERK1/2	85

2. Phenols				
Epicatechin-3-gallate (ECG)	A549, WI-38, MRC-5	10, 20 mg/kg	MMP-2, u-PA, E-cadherin, fibronectin, p-FAK	131
	BALB/c mice			
Epigalloccatechin-3-gallate (EGCG)	ARPE-19, HRMECs	1–100 μM	ROS, MMP-9, VEGF, VEGFR-2, PECAM/CD31	134
	BALB/c mice, SD rats	200 mg/kg		
	WPMY-1 prostate fibroblast cell	1.3–40 μM	ERK, AKT, RhoA, fibronectin	133
	DAOY medulloblastoma cell	10, 20 μM	β1 integrins	126
	4T1	100 μM	MicroRNA-16	181
	BALB/c mice	10 mg/kg	TAM infiltration, M2 polarization	
Resveratrol	primary culture chondrocytes	50μM	ECM, GAG, IL-1β	120
	HTB94	50 μM	p38, JNK, MMP-2/9, type II collagen, SOX-9, sulfated proteoglycan	128
	Chondrocyte	100 μM	MMP-3/9, COX-2, IL-1β, VEGF	121
	ELT-3-LUC, the primary cultures of human leiomyoma cells	10, 50, 100 µM	PCNA, α-SMA, fibronectin, Bax, Bcl-2, COL1A1, β-catenin	119
	Nude (Foxn1nu) mice	10 mg/kg		
	Hepa1-6, H22	10, 20, 40 µM	CD8+CD122+ Treg, TAMs/M2 macrophage	177
	C57BL/6 and BALB/c mice	50 mg/kg	STAT3,TGF-β1, IL-10, TNF-α, IFN-γ	
	THP-1, LM8	5, 10, 25, 50 µM	IL-10, MCP-1, TGF-β1	118
	C3H/He mice	10, 25 mg/kg	M2 macrophage	
	MCF-7, MDA-MB-231	50 µM	Cyclin D1, c-Myc, MMP-2/9, Sox2, Akt, STAT3, self-renewal signaling molecules	129
	KKU-213, KKU-100, CAFs	50, 100 µM	CAFs, IL-6	144
HS-1793	FM3A, THP-1	1.25, 2.5, 5 μM	M-2 phenotype TAM	178
	C3H/He mice	1.5 mg/kg	IFN-γ, VEGF, MMP-9, IL-6, IL-12, IL-10, TFG-β, IL-1β, TNF-α, CD206, CD204	
4,4′-dihydroxy-trans-stilbene	BALB/c 3T3 clone A31-1-1 cells, MCF-7	1, 2.5, 5, 7.5, 15, 30, 60, 90 µM	P53, p21, pRb, MMP-2/9, E-cadherin	130
Erianthridin	A549, H460	0–50 µM	Akt/mTOR/p70S6K, MMP-2/9	136
	CB17-Prkdcscid mice			
Hyperforin	Leukemic B-cells, HBMEC	0–5 µg/uL	MMP-9, VEGF	137
10-gingerol	HMT3522 S1, T4-2	0.25, 1, 2.5, 5, 10 µM	EGFR, β1-integrin	127
	nude mice			
Catechin	SD rats	25, 50 mg/kg	MMP-9, SOD, MDA, GSH	132
Ellagic acid	primary human chondrocytes	12.5, 25, 50 μM	iNOS, COX-2, NO, TNF-α, PGE2, IL-6, MMP-13, ADAMTS-5, collagen II, NF-κB, aggrecan	139
	C57BL/6 male wild-type (WT) mice	40 mg/kg		
Magnolol	HCC SK-Hep1 cells	50, 100 mg/kg	p-ERK, NF-κB p65, MMP-9, XIAP, CyclinD1, caspase-8, caspase-9	142
	BALB/cAnN.Cg-Foxn1nu/CrlNarl (nude) mice			
Procyanidins	HUVECs, A549 cells	0.75, 1.5, 3.1, 6.3, 12.5, 25, 50, 100 μg/ml	MMP-2, CD31	135
	Nude mice	10, 30 mg/kg		
Sesamin	A549, fibroblast cells	10 μM	α-SMA, E-cadherin, fibronectin, vimentin, p-Smad3, collagen I	124
	BALB/c mice	1, 10, 20 mg/kg		
Eugenol	CAF-64, CAF-180, MDA-MB-231, MCF-7	0.5, 1 μM	DNMT1/DNMT3A, E2F1	145
	nude mice			
Esculetin	LM8 cells	10, 50, 100, 200 μM	Cyclin-D1, CDK4, MMP-2, TGF-β1, VEGF, p-Stat 3, IL-10, MCP-1	140
	LM8-bearing mice	3, 10 mg/kg		
Fraxetin	LM8 cells	10, 50, 100, 200 μM	TGF-β1, p-Stat 3, IL-10, MCP-1	140
	LM8-bearing mice	3, 10 mg/kg		
Osthole	HTM cells	1, 3, 10, 30, 100 μM	FN, COL-IV, LN	125
	BALB/cJ mice	30 mg/kg		
4-Methylesculetin	rats	25, 50 mg/kg	MMP-3/9/13,ROS, GSH, H2O2, LPO, PCC, Thiol, e TNF-a, IL-1b, IL-6, COX-2 and PGE2	141
Daphnetin	MCs	0, 10, 20, 40 μM	Nrf2/keap1, Akt/NF-kB	122
Rosmarinic acid	Primary cultured chondrocytes	0, 25, 50, 75, 100 μg/mL	MMP-13, COX-2, ERK-1/2 and p38, PEG2	138
Gallic acid/Caffeic acid	EAT	40, 80 mg/kg	M1 macrophages	182
	mice		VEGF, MMP-2/9, COX-2	
zingerone	RHE tissues	50 µg/ml	MMP-1/3/12, NOTCH1, MAML3, CTSV, PMAIP1, ARG2	143
Acetyl zingerone	RHE tissues	50 µg/ml	MMP-1/3/12, COL11A2, VCAN, SPARC, NOTCH1, MAML3, CTSV, PMAIP1, ARG2	143

3. Polysaccharides, Saponins and Terpenoid				
Astragalus polysacharin	MHCC97H, Huh7, THP-1	8, 16 mg/ml	CD68, CD86, CD206	184
	HCC mice	50, 100, 200 mg/kg		
Polyphyllin II	DEN-induced pulmonary adenoma rats	50 mg/10ml/kg	MMP-9, TIMP-2	148
Ginsenoside Rh2	RAW267.4, THP-1, A549, H1299	60, 100, 140 µM	VEGF, MMP2/9, CD206, CD16/32,TNFα, iNOS, Arg-1	186
Dioscin	B16, RAW264.7	1, 2 µM	E-Cad, N-Cad, Cx43, CD206, IL-6, TNFα, IL-1β	187
	Melanoma metastasis model	30, 60 mg/kg		
Astragaloside IV	GCAFs, BGC-823	10, 20, 40 µM	Pan-CK, E-cadherin, Vimentin, PDGFR-β, α-SMA	149
			miR-214/301a	
Andrographolide	GBM8401, U251	5, 10, 20, 40 µM	MMP-2, ERK1/2, Raf, MEK, JNK,CREB, c-Jun, c-fos, SP-1	153
Hinokitiol	B16-F10, 4T1	1.25, 12.5, 125, 1250 nM	Akt, ERK	150
	Melanoma/mammary carcinoma metastasis model			
	B16-F10	1, 2, 5 µM	MMP-2, MMP-9	151
Artemisinin	THP-1	20, 40, 80 μg/mL	EMMPRIN, MMP-9, JNK, p38, ERK	152
Triptolide	4T1, RAW264.7, THP-1	5, 10, 20 nM	CD206, Arg-1, IL-10, CD68, MCP-1, CD163	190
	Colon tumor model	0.05, 0.2 mg/kg		
	Tumor syngeneic graft model			
Oleanolic acid	U373, THP-1	20, 30 µM	STAT3, JAK	191, 192
Corosolic acid	U373, macrophage	10, 20, 30 μM	STAT3, CD163, IL-10	192
Paeoniflorin	LLC	1, 3, 10, 30, 100 μM	CD11c, F4/80	189
	LLC injected mice			
Asiatic Acid	keloid fibroblasts	3, 10, 30 μM	PPAR-γ, PAI-1, Smad	154

4. Alkaloids and Quinones				
α-Chaconine	BAECs	1, 2, 3 μg/mL	JNK, PI3K, NF-κB, Akt, MMP-2	155
α-Tomatine	MCF-7	0.5, 0.75, 1 µM	MMP-2/9, PKCα, ERK1/2, NF-κB	157
α-Solanine	A2058, A375	4.6, 9.2, 13.8, 18.4 µM	MMP-2/9, ERK, JNK, NF-κB, PI3K, Akt	156
Berberine	Type 2 diabetic animal models (rat)	200mg·kg^−1^·day^−1^	IGF-1R, MMP-2/9/14, ERK, a-SMA, Collagen I	161
	THP-1	5, 10, 25, 50 μM	MMP-9, EMMPRIN, p38	162
Aristolochic acid	TIB-202, THP-1	1, 5, 10, 20 μM	NF-κB, MMP-9	59
Solasodine	A549	4, 8, 12 μM	MMP/9, EMMPRIN, RECK, TIMP-1/2, PI3K, Akt, miR-21	163
Evodiamine	MDA-MB-23	15, 30, 60, 90, 120 μM; 10 mg/kg	Bcl-2, Bax, P27, CDK6, Cyclin D1, MMP-9, ERK, p38, JNK	164
	MDA-MB-23 injected mice			
Oxymatrine	nucleus pulposus cells	0.25, 0.5, 1, 2 mg/mL	HMGB1, TLR4, IkBa, NF-κB	160
	IDD rat			
Emodin	EO771, 4T1, MCF7, MDA-MB-231, TAMs	40 mg/kg	TGF-β1, AKT, STAT3, ZEB1, Snail, Twist, Smad2/3/4	193
	Breast cancer model (mice)			
	EO771, 4T1	25, 50 μM	JMJD3, IRF4, STAT6, H3K27m1/2/3	194
	Breast cancer model (mice)	40 mg/kg		
	hSf, hDF	1, 2, 3, 4 μM	LOXL1	167
	ICR female mice	1 mg/L		
Shikonin	liver fibrosis C57 mice	2.5, 5 mg/kg	α-SMA, Beclin-1, LC3 I/II, TGF-β1, Smads	166
Thymoquinone	liver fibrosis Kunming mice	20, 40 mg/kg	Collagen I, α-SMA, TIMP-1, TLR4, PI3K, MAPK, LKB1	165

**TABLE 2:** Structural formula of the Natural products.

1. The structural formula of flavonoids-1
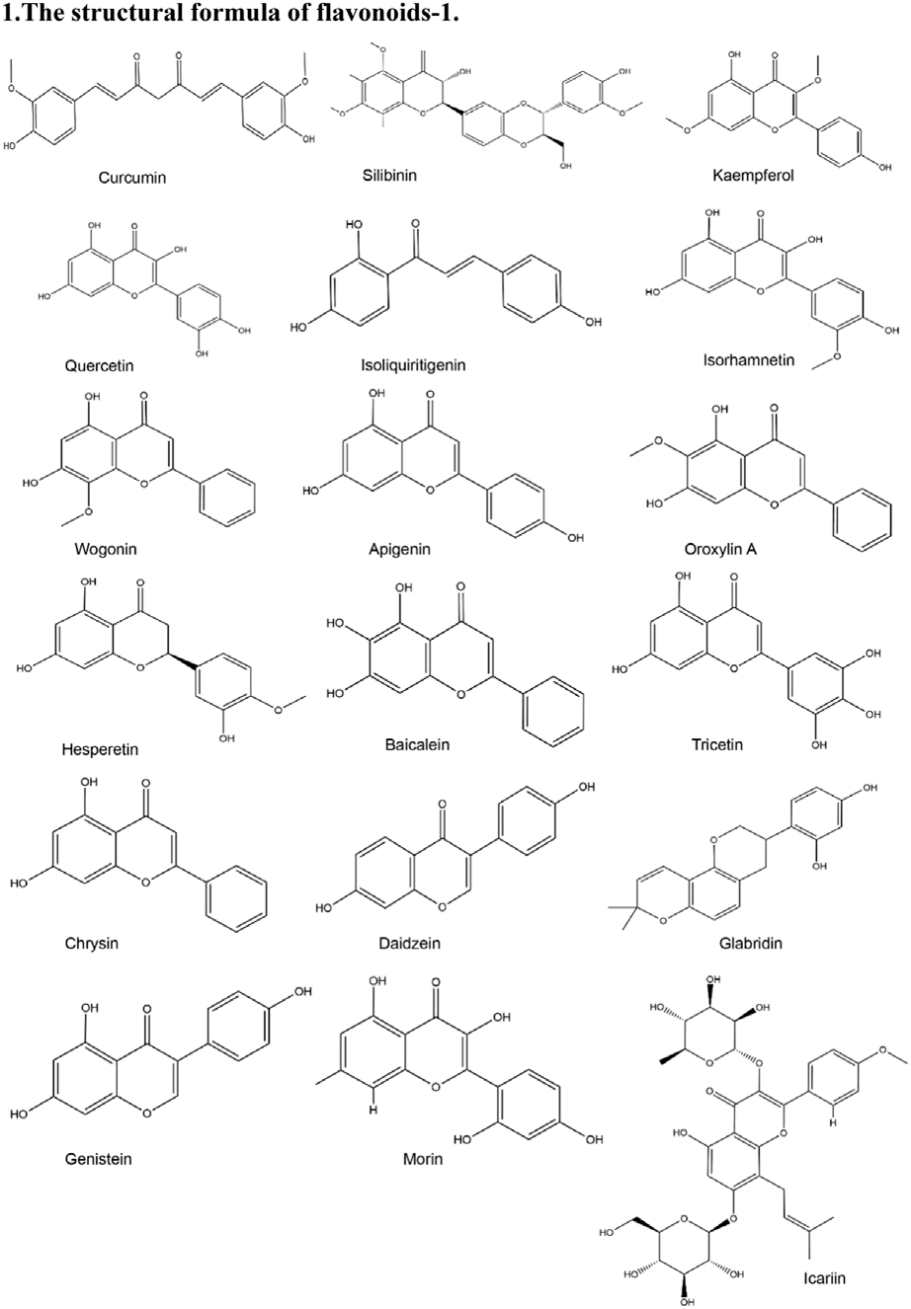
2. The structural formula of flavonoids-2
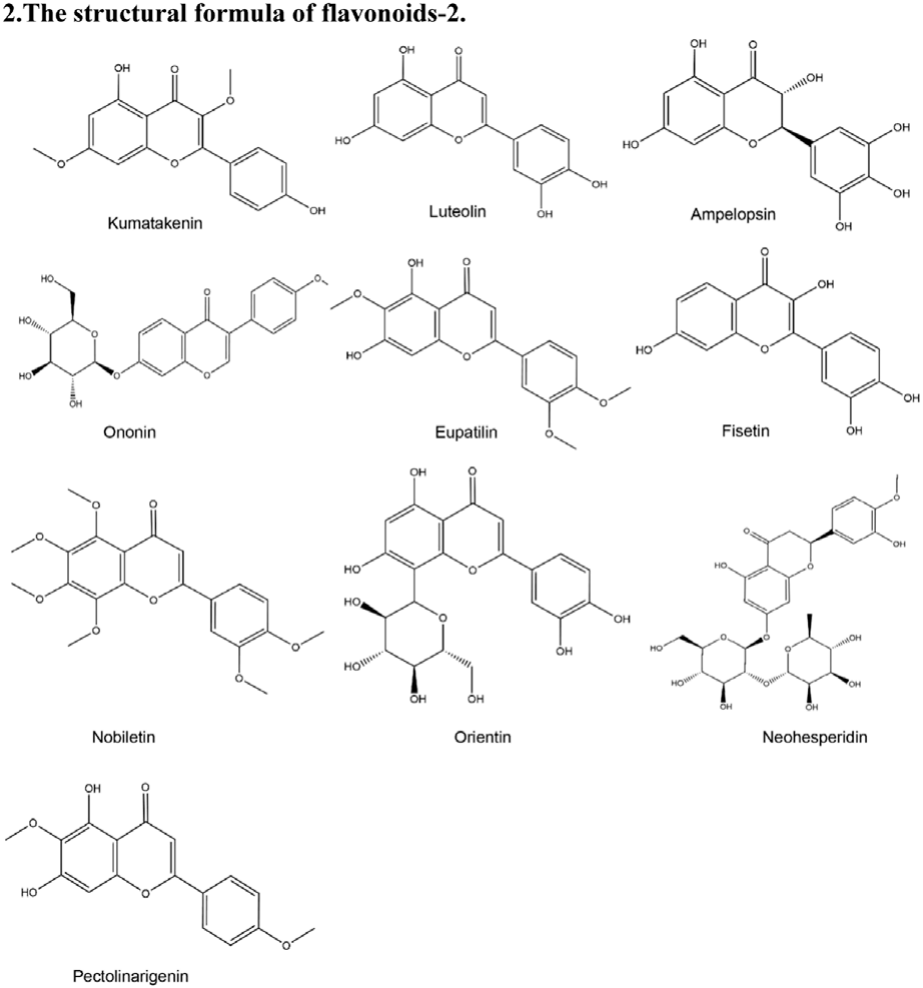
3. The structural formula of phenols-1
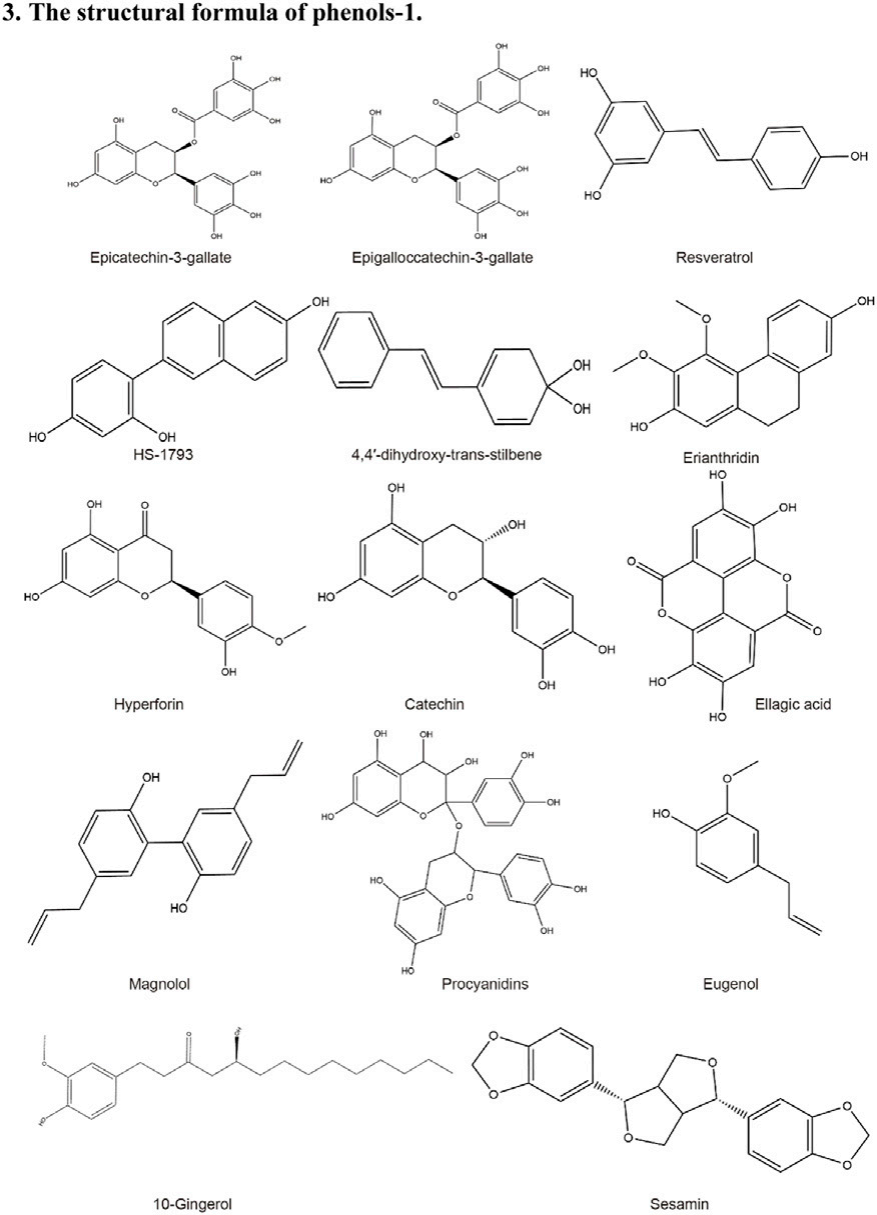
4. The structural formula of phenols-2
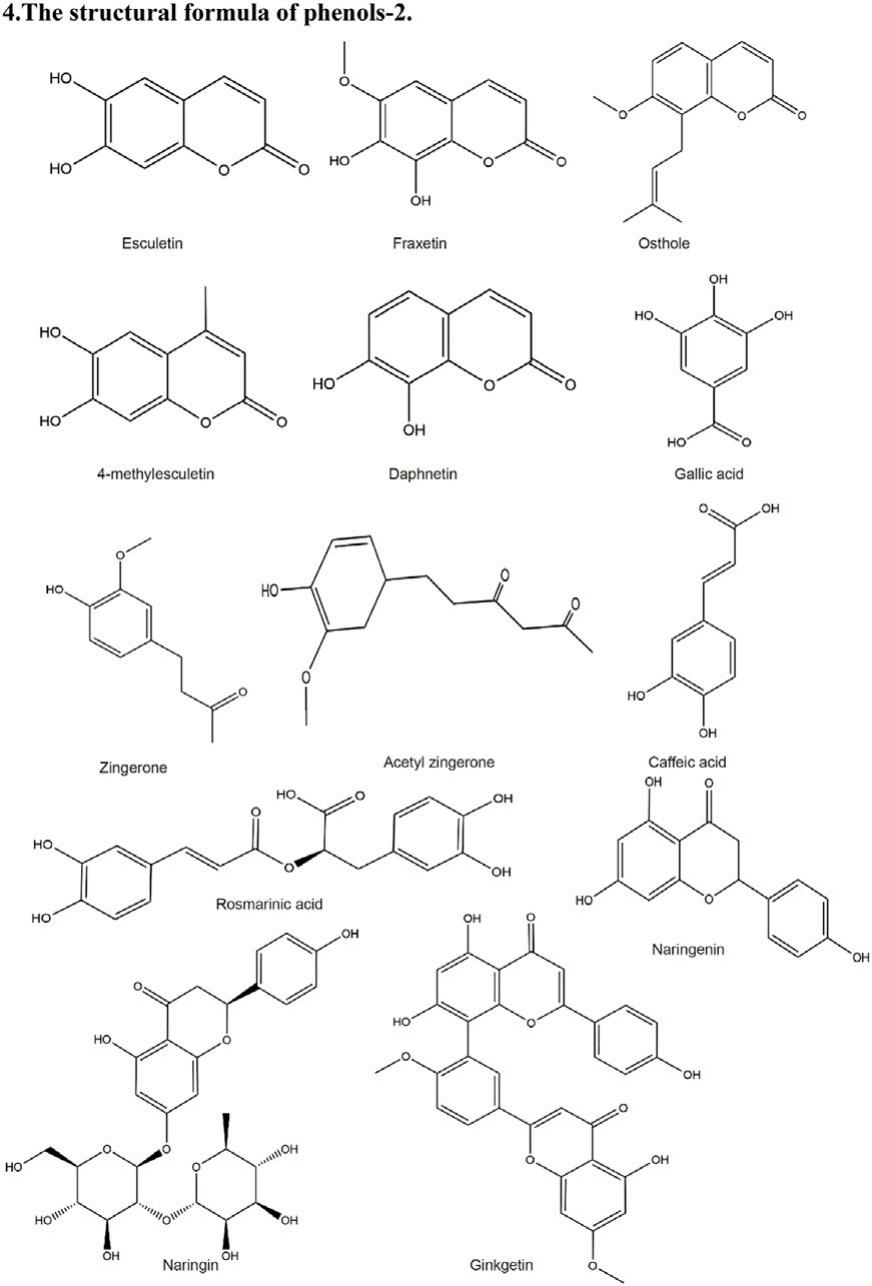
5. The structural formula of polysaccharides, saponins and terpenoid
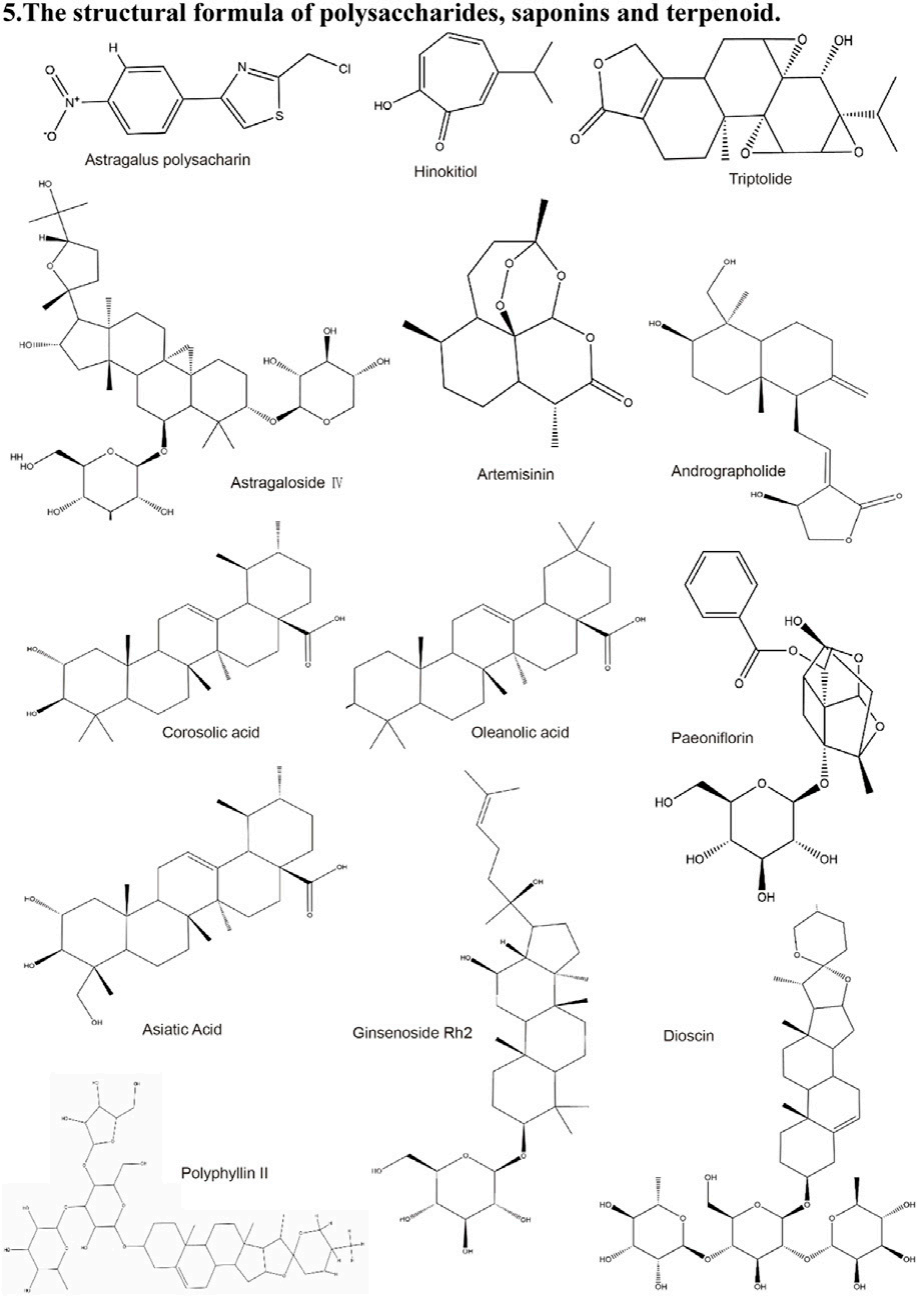
6. The structural formula of alkaloids and quinones
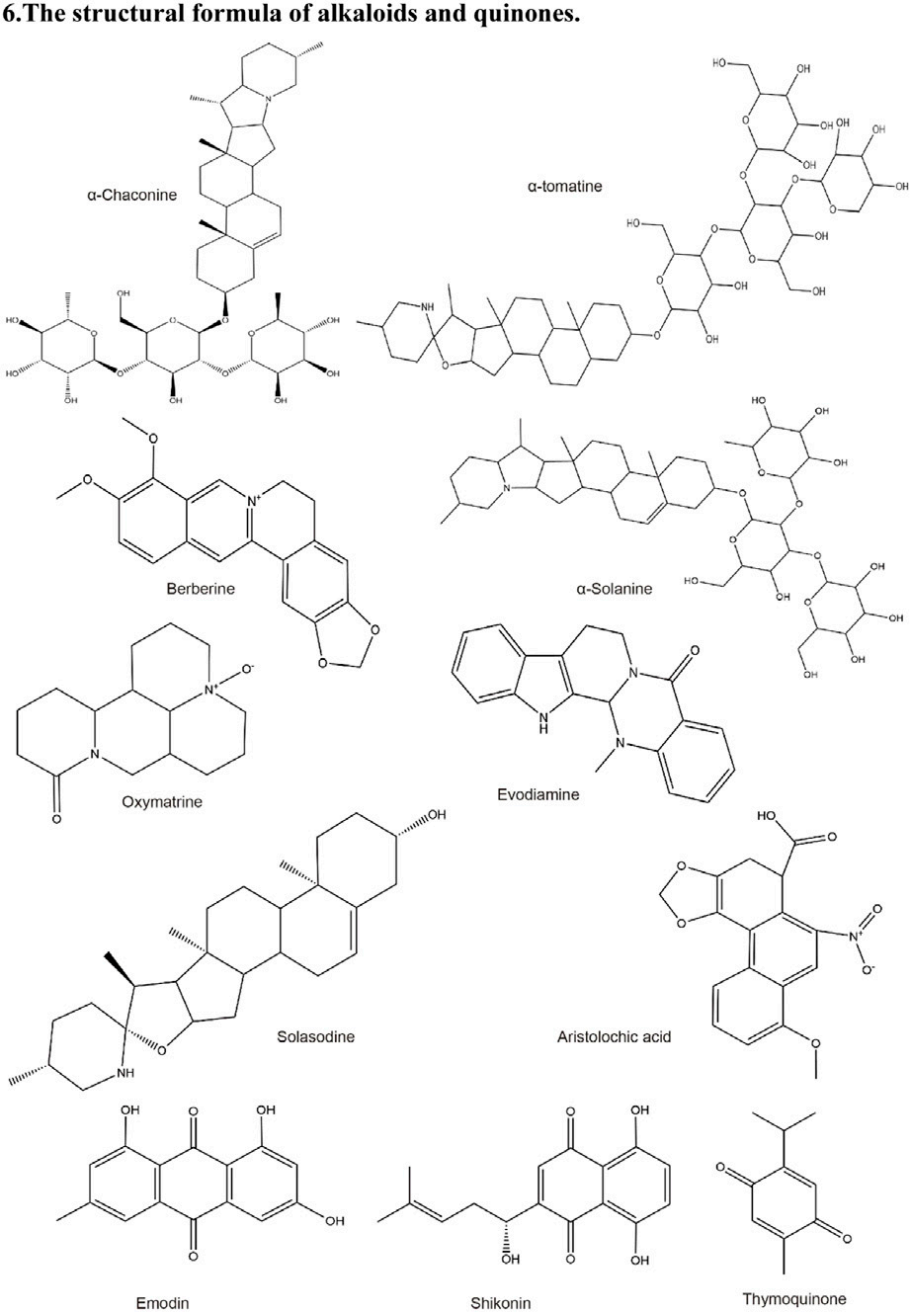

## 5 Discussion and outlook

**FIGURE 3:**
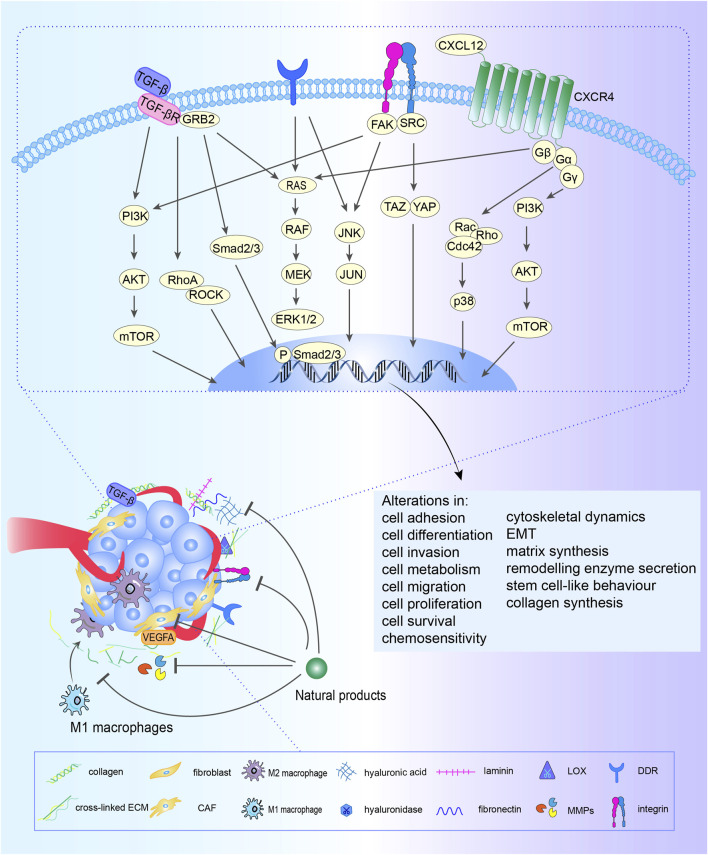
Natural products target ECM. Natural products normalize ECM via reducing deposition, altering modification and inhibiting degradation of ECM. Existing research suggests that TGF-β, MMPs, VEGFA, integrins, DDR and so on, would be a drug target. These signaling pathways are intertwined. Simultaneously, flavonoids, phenols, polysaccharides, and saponins, etc. Could regulating ECM by triggering variations about cell biological behavior and phenotype. In addition, natural products also affect the macrophage polarization.

Compared to traditional approaches that target tumor cells, strategies of targeting ECM components and tumor stromal cells may have several potential advantages (Bonnans et al., 2014). First, the components of ECM and stromal cells is more stable in the genetic and therefore it is less likely to acquire resistance to cytotoxic agents. Second, many solid tumor malignancies possess common alterations in TME. Thus, approaches targeting the TME can be broadly applied to different tumor types. With the rapid development of natural product researches, the active ingredients therein have shown therapeutic effects in the clinical with fewer side effects. By means of collating natural products, we found that they showed great potential intervention on the matrix stiffness formed by the change of components and receptors during ECM remodeling, and the crosstalk between CAFs, TAMs and cancer cells. Flavonoids, polyphenols, alkaloids, terpenes and quinones mainly target the deposition and degradation of ECM, with collagen, fibronectin, integrins and MMPs as the main targets, while polysaccharides mainly target TAMs (Figure 3).

Collagen and HA are the main factors determining matrix stiffness during the ECM deposition mechanism (Huang et al., 2021a). Fibronectin also plays a vital role during malignant transformation. Reducing collagen synthesis can lessen ECM stiffness, and alleviate malignant fibrosis hyperplasia. Prompting collagen rupture can facilitate the penetration of many conventional chemotherapeutic agents and nanoparticles through the barrier of the hardened matrix in the TME. Among the polyphenolic compounds from nature, curcumin, resveratrol, eupatilin, isoliquiritigenin and morin can significantly reduce collagen, laminin and fibronectin deposition in Phase I of the management of cancer. Considering TGF-β′s crucial role during collagen synthesis, TGF-β signaling is the most promising target to inhibit collagen synthesis (Liu et al., 2012; Chauhan et al., 2013). There are multiple drugs targeting TGF-β being evaluated in clinical trials. Flavonoids such as ampelopsin, pectolinarigenin, neohesperidin, apigenin and other polyphenolic compounds such as sesamin, osthole and daphnetin can reduce collagen deposition by inhibiting TGF-β signaling. There are also small molecules targeting the TGF-β receptor (TGF-βR) that are widely used in cancer treatment trials, but they lead to severe systemic toxicity due to instability and low specificity (Tanaka et al., 2010; DaCosta Byfield et al., 2004). Garcimultiflorone K inhibits TGF-β signaling by down-regulating TGF-βR, showing no apparent toxicity. In consideration of the complex roles of TGF-β and TGF-βR in inflammation and tumorigenesis, a comprehensive understanding of tumor traits, stages of disease and TME is necessary for researchers to be cautious with therapeutic targets involving TGF-β and TGF-βR. Two therapeutic strategies for HA are also under investigation, including inhibition of HA synthesis and enhancement of HA degradation (Whatcott et al., 2011). Researches on fibronectin mainly focused on its application as a target for precise drug delivery, several therapies as targeting fibronectin EDA and extra domain B (EDB) have been developed in an attempt to inhibit tumor neovasculature (Castellani et al., 1994). In addition, ECM is the primary target of dicarbonyl (Zhou et al., 2015). For example, methylglyoxal (MG) is a highly reactive dicarbonyl group that interacts with proteins to form advanced glycation end products (AGEs), favoring ECM stiffness (Nowotny et al., 2018). Collagen is the primary target of MG modification in the ECM *in vivo*. Some natural products such as EGCG, phloridzin, as well as for quercetin, have been reported possess the trapping capacity of dicarbonyls (Li et al., 2014; Hung et al., 2018; Zhao et al., 2022).

During the Phase II, ECM modification stage, drugs can target matrix stiffness sensors such as integrins, DDR1, CD44-HA and RHAMM-HA interactions to reduce the generation of abnormal ECM structures and soften hardened ECM. Integrins can bind to various proteins, such as collagen, fibronectin, and laminin, triggering mechano-transduction and carcinogenesis (Xiong et al., 2021). Integrin inhibitors such as Vitaxi and volociximab can inhibit disease progression based on preclinical studies, showing therapeutic potential in various cancers (Posey et al., 2001; Ricart et al., 2008; Bell-McGuinn et al., 2011). Since many integrins are also expressed in immune cells, the impact of integrin inhibitors on immune surveillance needs to be considered (Su et al., 2016). Among natural products, only a few phenolic compounds can modulate integrins, such as silibinin, EGCE and [10]-gingerol, suggesting that more research should focus on integrins. Emodin can enhance the effect of LOX1, but no evidence has been observed for natural products to reverse abnormal ECM modification, and more researches should be conducted on the effects of natural products on ECM modifying enzymes and substrate stiffness sensors. Combinations of DDR1 inhibitors and classical chemotherapeutics reduce tumor burden in orthotopic xenograft and orthotopic pancreatic cancer models (Aguilera et al., 2017). If natural DDR inhibitors can be found and combined it with chemotherapeutic drugs, the clinical effect can be enhanced while the occurrence of adverse reactions can be reduced. CD44 is mainly functions receptor for HA, collagen, fibronectin, and growth factors. However, due to the lack of a comprehensive understanding of all CD44 isoforms and the consequences of knocking out a mixture of CD44 isoforms, some challenges remain for the clinical application of targeting CD44. Several therapeutic approaches targeting the RHAMM-HA interaction are under evaluation in preclinical studies in various cancers. Targeted collagen cross-linking can significantly reduce ECM stiffness in cancer (Huang et al., 2021a). LOX is a core target that catalyzes the covalent cross-linking and matrix hardening of collagen and elastin. Pan-LOX family inhibitors and specific inhibitors of a LOX family member have been developed. These LOX inhibitors have shown anticancer effects in preclinical studies, but long-term use can cause aortic damage and osteoporosis, limiting their clinical application (Chen et al., 2012). Currently there are not enough studies to find out the effect of natural products on LOX, thus further research can be done in this field.

In terms of regulating ECM degradation, studies on the intervention of MMPs by natural products are the most abundant. Most flavonoids have inhibitory effects on MMPs, especially MMP-2/9, to reduce ECM degradation and anti-angiogenesis in Phase III. For example, silibinin, oroxylin A, kaempferol, wogonin, baicalein, chrysin and daidzein target the ERK pathway and inhibit MMP-2/9-mediated ECM degradation and cell motility. Combining natural products with ERK inhibitors may be a good strategy to reduce cancer cell invasion. Hesperetin and curcumin can target the TGF-β/Smad pathway to reverse the degradation of ECM. Further research can focus on natural products targeting TGF-β or combining with TGF-β inhibitors. Since collagen degradation releases growth factors and cytokines in the ECM, triggering a cascade of inflammatory signals and tumor progression, inhibition of ECM degradation could theoretically reduce cell invasion. However, MMPs degrade collagen and reduce the stiffness of the matrix, and also help to deliver drugs more efficiently into solid tumors, so the optimal timing for the application of such therapy should be carefully considered. Furthermore, MMPs have pleiotropic activity, for example, MMP-8 has both pro-tumor and anti-tumor functions. MMPs may have opposite functions at different stages of the disease (Zeisberg et al., 2006). The poor efficacy or even negative effects of MMPs inhibitors in clinical trials may be due to the inappropriate application of broad-spectrum inhibitors, selection of patients with advanced metastatic disease, inadequate/insufficient evaluation of the therapeutic window, and reliance on monotherapy (Juurikka et al., 2019). Some natural products that simultaneously regulate the levels of MMPs and TIMPs to maintain the balance deserve further study to discover modulators of MMPs adapted to complex TAM. Some compounds showed no cytotoxicity when reducing ECM degradation by targeting the p38 MAPK pathway, providing ideas for the study of new drugs that target the ECM while protecting normal tissues. Regulating multiple targets is an advantage of natural products. For example, some compounds can increase normal collagen production while inhibiting MMPs (such as quercetin, glabridin, resveratrol, acetyl zingerone, ellagic acid, and rosmarinic acid) or decrease angiogenesis (such as EGCG, hyperforin, Esculetin, and fraxetin). The ability of EGCG and luteolin to synergistically target TGF-β and myofibroblasts suggests that the combined use of these compounds may enhance ECM targeting. Making adjustment based on the original structure of natural products can significantly increase the activity and improve the stability of the natural product, such as the synthetic resveratrol analog 4,4′-dihydroxy-trans-stilbene and the designed acetyl zingerone on the zingerone structure.

**FIGURE 4:**
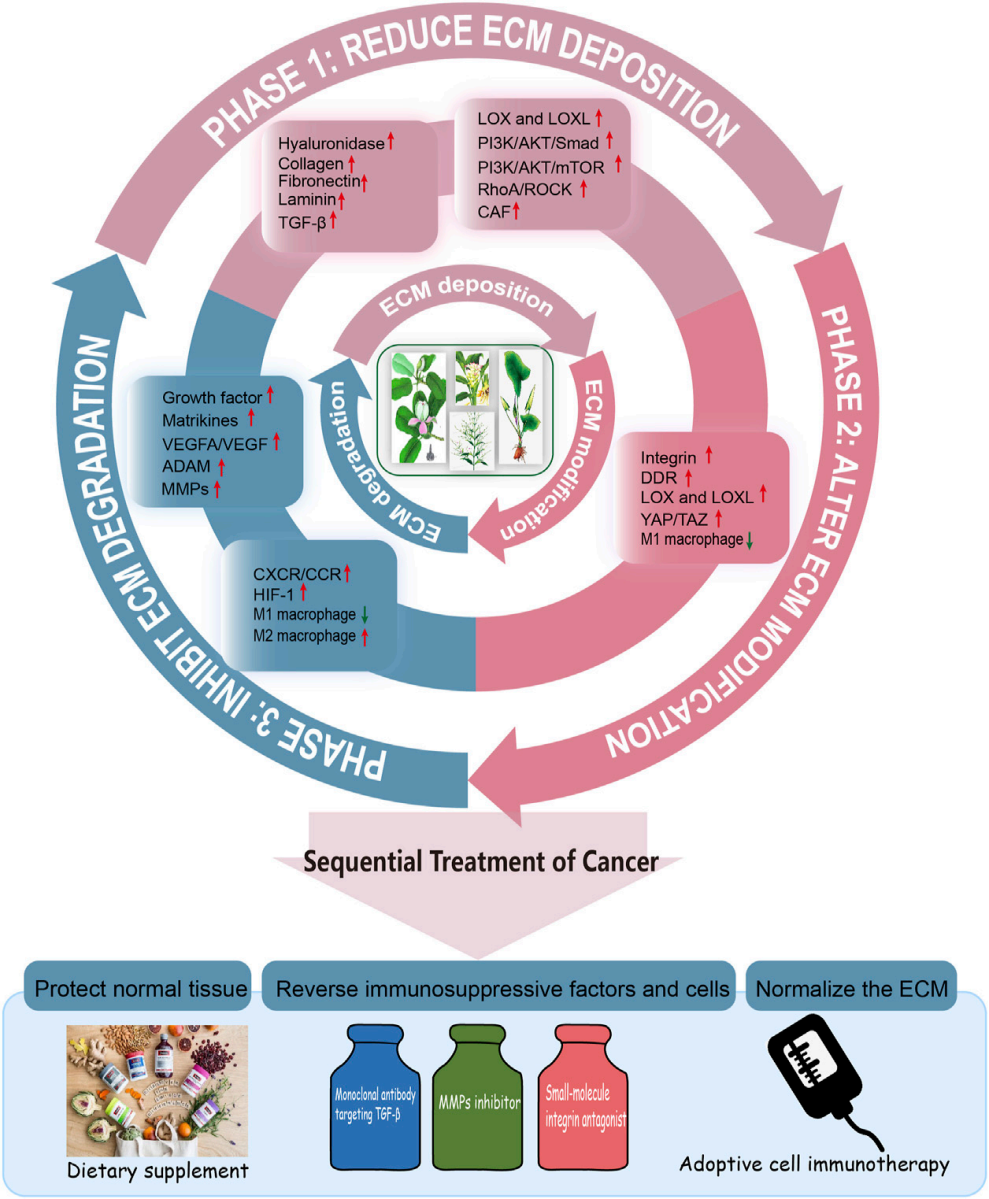
The whole process management of cancer. Efforts to establish a whole-process management of cancer treatment is critical for global cancer control. Thus, we put forward the concept of three phases of the whole-process management of cancer with ECM remodeling. Phase I: Natura products reduce ECM deposition. Phase II: Natura products alter ECM modification. Phase III: Natura products regulate degradation of ECM. Therefore, the sequential treatment of cancer should be constructed to normalize the ECM, protect the normal tissue, reverse immunosuppressive factors and cells.

The combination of three phases sequential cancer therapy and the ECM remodeling trilogy makes it possible to manage cancer throughout the entire process (Figure 4). In the vicious circle of ECM remodeling that drives cancer progression, some natural products with bidirectional regulatory effects may have great therapeutic potential. How to grasp the optimal treatment window during ECM dynamic changes and tumor progression is the focus of further exploration. Overall, ECM is a promising direct therapeutic target in cancer treatment (Jiang et al., 2022), but Gordian Knots still exists in the field of scientific research and clinical practice. There is a lack of material that can accurately mimic ECM *in vitro*, and the detailed ECM components in various disease contexts remain unclear, as are the specific targets of different proteases. Given its importance in disease, further studies/researches should focus on a detailed biological understanding of ECM morphology and its components such as key enzymes, its structure and signaling molecules, therefore to inspire the identification of next-generation cancer diagnostic and therapeutic targets.

## Figures and Tables

**Table:** 

**TME**	tumor microenvironment
**ECM**	extracellular matrix
**CAFs**	cancer-associated fibroblasts
**TAMs**	tumor-associated macrophages
**HA**	hyaluronic acid
**MMPs**	matrix Metalloproteinases
**HSPGs**	heparan sulfate proteoglycans
**TGF-β**	transforming growth factor beta
**CXCL12/10**	C-X-C motif chemokine ligand 12/10
**CXCR4/3**	C-X-C motif chemokine receptor 4/3
**CCR9**	C-C motif chemokine receptors 9
**CCL21/25**	C-C motif chemokine ligand 21/25
**MCP-1**	monocyte chemoattractant protein-1
**CSF-1**	colony stimulating factor-1
**IL-6**	interleukin-6
**PGE2**	prostaglandin E2
**α-SMA**	α-smooth muscle actin
**FGF-2**	fibroblast growth factor-2
**PDGF**	platelet-derived growth factor
**EGF**	epidermal growth factor
**LOX**	lysyl oxidase
**LOXL**	LOX-like protein
**IGF**	insulin-like growth factor
**HAPLN1**	hyaluronan and proteoglycan-linked protein 1;
**HMM-HA**	High molecular weight HA
**LMM-HA**	low-molecular-mass HA
**PLODs**	procollagen-lysine 1, 2-oxoglutarate 5-dioxygenases
**TG2**	tissue transglutaminase 2
**ADAMs**	disintegrin and metalloproteinases
**IGFBP-3**	insulin-like growth factor-binding protein 3
**VEGF-A**	vascular endothelial growth factor-A
**TIMP**	tissue inhibitor of metalloproteinases
**GF**	growth factors
**MT1-MMP**	membrane type-1 matrix metalloproteinase
**AP-1**	activator protein-1
**JAK2**	Janus kinase 2
**PCA**	prostate cancer
**FAK**	focal adhesion kinase
**u-PA**	urokinase-type plasminogen activator
**GBM**	glioblastoma multiforme
**SP-1**	Specificity protein-1
**Col X**	collagen X
**Nrf2**	nuclear factor-erythroid 2related factor 2
**ECG**	Epicatechin-3-gallate
**EGCG**	Epigalloccatechin-3-gallate
**GCAFs**	gastric cancer-associated fibroblasts
**PAI-1**	plasminogen activator inhibitor-1
**EMMPRIN**	extracellular matrix metalloproteinase inducer
**TLR4**	toll-like receptor 4
**CDK6**	cyclin-dependent kinase 6
**SFDA**	State Food and Drug Administration of China
**PD-L1**	programmed death ligand-1
**PD-1**	programmed death-1
**TGF-βR**	TGF-β receptor
**EDB**	extra domain B
**DDR1**	discoid domain receptor 1
